# Simultaneous reduction of all ORMDL proteins decreases the threshold of mast cell activation

**DOI:** 10.1038/s41598-023-36344-5

**Published:** 2023-06-14

**Authors:** Livia Demkova, Viktor Bugajev, Pavol Utekal, Ladislav Kuchar, Björn Schuster, Petr Draber, Ivana Halova

**Affiliations:** 1 Laboratory of Signal Transduction, Institute of Molecular Genetics of the Czech Academy of Sciences, Videnska 1083, 14220 Prague 4, Czech Republic; 2 Research Unit for Rare Diseases, Department of Paediatrics and Inherited Metabolic Disorders, First Faculty of Medicine, Charles University and General University Hospital in Prague, Prague, Czech Republic; 3 Czech Centre for Phenogenomics, Institute of Molecular Genetics of the Czech Academy of Sciences, Prague, Czech Republic; 4 CZ-OPENSCREEN, Institute of Molecular Genetics of the Czech Academy of Sciences, Prague, Czech Republic

**Keywords:** Signal transduction, Lipid signalling, Sphingolipids, Mast cells

## Abstract

In mammals, the ORMDL family of evolutionarily conserved sphingolipid regulators consists of three highly homologous members, ORMDL1, ORMDL2 and ORMDL3. *ORMDL3* gene has been associated with childhood-onset asthma and other inflammatory diseases in which mast cells play an important role. We previously described increased IgE-mediated activation of mast cells with simultaneous deletions of ORMDL2 and ORMDL3 proteins. In this study, we prepared mice with *Ormdl1* knockout and thereafter, produced primary mast cells with reduced expression of one, two or all three ORMDL proteins. The lone deletion of ORMDL1, or in combination with ORMDL2, had no effect on sphingolipid metabolism nor IgE-antigen dependent responses in mast cells. Double ORMDL1 and ORMDL3 knockout mast cells displayed enhanced IgE-mediated calcium responses and cytokine production. Silencing of ORMDL3 in mast cells after maturation increased their sensitivity to antigen. Mast cells with reduced levels of all three ORMDL proteins demonstrated pro-inflammatory responses even in the absence of antigen activation. Overall, our results show that reduced levels of ORMDL proteins shift mast cells towards a pro-inflammatory phenotype, which is predominantly dependent on the levels of ORMDL3 expression.

## Introduction

Mast cells are a heterogeneous population of immune cells derived from myeloid haematopoietic progenitors in the bone marrow of vertebrates. They circulate as precursors and migrate into tissues, where they differentiate and undergo maturation^1^. Mature mast cells reside in connective tissues and mucosal surfaces in close proximity to blood vessels^2^. Thus, together with antigen-presenting leucocytes, they are often the first immune cells in contact with the external environment^2,3^. Based on their local microenvironment, mast cells are capable of producing a variety of bioactive mediators in response to multiple stimuli^3,4^. Their anatomical distribution and relative plasticity allows mast cells to play a central role in the regulation of both innate and adaptive immune responses, as well as influence the phenotype and function of non-immune cell types^1,2^. The diverse interactions and responses mediated by mast cells have implicated them in many diseases, such as atopy, asthma and parasitic infections^1^.

One class of bioactive mediators that are both produced by and stimulate mast cells are sphingolipids^5,6^. Sphingolipids are a diverse group of lipids that share a common sphingoid backbone consisting of an L-serine headgroup and a fatty acyl moiety^7,8^. In cells, sphingolipids fulfill important structural roles by being incorporated into membranes. In addition to their mechanical role, as bioactive lipids they also participate in multiple cellular signalling networks^9^. De novo sphingolipid synthesis starts in the endoplasmic reticulum (ER) with the condensation of L-serine and a fatty acyl-coenzymeA (CoA), typically palmitoyl-CoA. The reaction is catalysed by serine palmitoyltransferase (SPT), the rate-limiting enzyme in de novo synthesis^7,8^. The pathway proceeds through a number of intermediate products, until the generation of ceramides, which can further be processed in the Golgi apparatus to form more complex sphingolipids. These complex sphingolipids undergo degradation back into ceramides via the salvage pathway^7^.

ORMDL proteins belong to a family of evolutionarily conserved small, transmembrane proteins localised to the ER membrane^10^. One of their main known functions is inhibition of the SPT enzyme and thereby, de novo sphingolipid biosynthesis^11–14^. Vertebrates encode three paralogous ORMDL proteins, ORMDL1, 2 and 3, which share around 80% amino acid identity in humans and 95% identity with murine orthologues^10^. Genome-wide association studies of asthmatic patients and health-matched controls revealed that single nucleotide polymorphisms (SNPs) located in the locus, where *ORMDL3* resides, are associated with childhood-onset asthma^15,16^. As such, ORMDL3 has been the focus of extensive research, with fewer studies dedicated to exploring the functions of the other two paralogs.

Mast cells are known to play a key role in the pathophysiology of asthma^17^. Investigating the role of not only ORMDL3, but also the highly homologous ORMDL1 and 2 proteins in these immune cells can contribute to the understanding of the aetiology and potential treatment of the respiratory disease. We previously showed that bone marrow-derived mast cells (BMMCs) and peritoneal-derived mast cells with reduced levels of ORMDL3 exhibited enhanced transcription and secretion of pro-inflammatory cytokines and eicosanoids following antigen stimulation^18–20^. The sole removal of ORMDL2 (O2KO) did not affect mast cell physiology. However, deletion of ORMDL2 in combination with ORMDL3 potentiated the changes that were observed when ORMDL3 was knocked out (O3KO)^19^.

In the present study, we explored the interactions and redundancy of individual ORMDL proteins in mast cells. Using combined genome-editing techniques with RNA interference, we prepared primary mast cells with reduced expression of one, two or all three ORMDL proteins. We investigated the effects that reduced levels of ORMDL proteins have on mast cell functions. Confirming our previous findings, we demonstrated that ORMDL3 is the dominant ORMDL paralog responsible for maintaining sphingolipid homeostasis, as well as regulating IgE-mediated responses. Additional reduction of ORMDL1 or 2 potentiated the phenotypes associated with ORMDL3 deficiency. Mast cells with reduced levels of all three ORMDL proteins exhibited pro-inflammatory responses even in the absence of antigen activation. Altogether, the results of this study indicate that reduced levels of ORMDL proteins prime mast cells and reduce their activation threshold.

## Results

### Preparation and characterisation of BMMCs with ORMDL1 KO

In our previous study, we prepared single O2KO and O3KO mice, as well as mice with concurrent deletions of ORMDL2 together with ORMDL3 (O2/3dKO)^19^. BMMCs derived from O2/3dKO mice exhibited a reduction but not complete loss of total ORMDL proteins^19^, indicating that all three ORMDL paralogs are expressed in BMMCs. As the role of ORMDL1 in mast cell physiology has not yet been determined, we prepared O1KO mice using CRISPR-Cas9 gene editing (Supplementary Fig. S1a, b). Subsequently, we generated O1/2dKO and O1/3dKO mice by crossbreeding of the respective single KO mice. Grossly, O1/3dKO mice were significantly smaller in size and weighed less than their littermates (Supplementary Fig. S1c). No significant morphological differences were noted between O1/2dKO and age-matched wildtype (WT) mice (Supplementary Fig. S1c).

**Figure 1:**
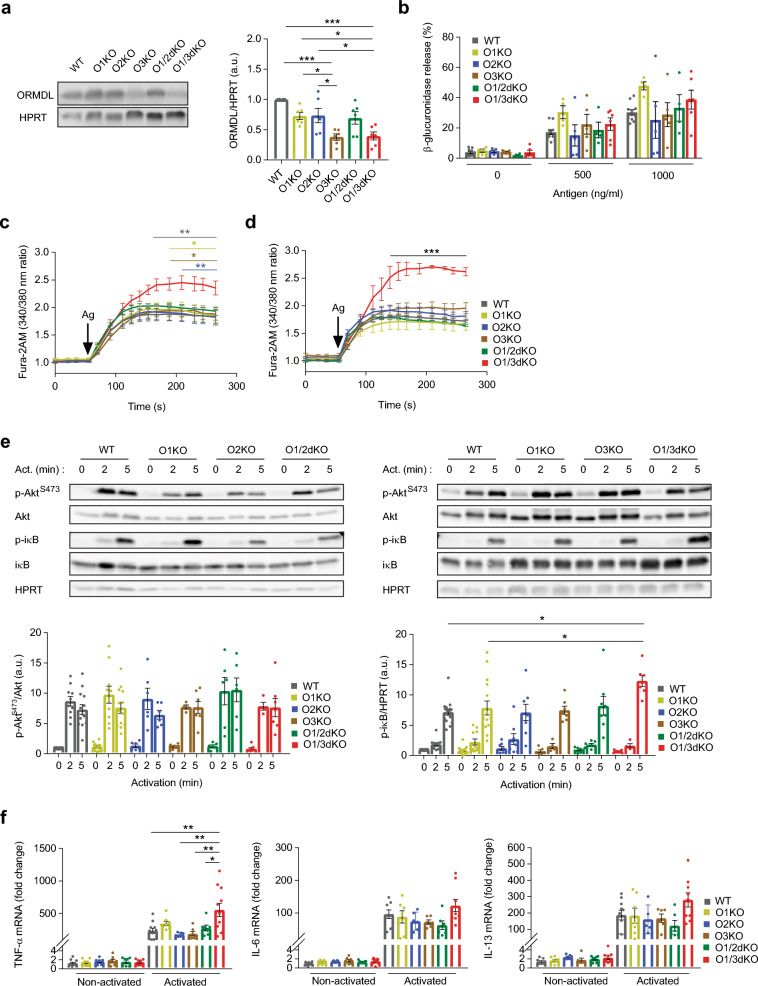
Impact of single and double ORMDL KOs on the activation of BMMCs. (**a**) Representative immunoblot and statistical analysis of total ORMDL protein levels in BMMCs derived from the indicated WT and transgenic mice. HPRT was used as a loading control. n = 6 biological replicates. Uncropped immunoblots are shown in Supplementary Fig. S5. (**b**) Release of β-glucuronidase as a measure of degranulation in BMMCs activated with 500 ng/ml and 1000 ng/ml TNP-BSA antigen. n = 4–11 biological replicates. Fura-2AM fluorescence as a measure of intracellular calcium at baseline and following activation with (**c**) 500 ng/ml TNP-BSA antigen and (**d**) 1000 ng/ml TNP-BSA antigen. Black arrows indicate times at which activators were added to cells. n = 4–11 biological replicates. (**e**) Immunoblot analysis with statistical evaluation of p-Akt, p-iκB and corresponding loading controls from lysates of non-activated BMMCs and BMMCs activated with 1000 ng/mL TNP-BSA antigen for 2 and 5 min as indicated. n = 4–14 biological replicates. Uncropped immunoblots are shown in Supplementary Figs. S6 and S7. (**f**) Relative mRNA expression of TNF-α, IL-6 and IL-13 in non-activated BMMCs and BMMCs activated with 1000 ng/ml TNP-BSA antigen for 1 h. n = 6–11 biological replicates. Data were analysed by one-way ANOVA with Tukey’s posttest (**a**, **b** and **f**), two-way ANOVA with Tukey’s posttest (**c** and **d**) and Kruskal–Wallis test with Dunn’s posttest (**e**). *p < 0.05, **p < 0.01 and ***p < 0.001. In (**c**), colour of lines and asterisks indicate statistical significance of O1/3dKO versus corresponding phenotypes—WT = grey, O1KO = yellow, O2KO = blue and O3KO = brown. In (**d**), black line and asterisks indicate statistical significance of O1/3dKO cells versus all other samples. All the results are represented as mean ± SEM. Ag—Antigen, Act.—Activation.

As part of our current study, we derived BMMCs from WT mice and mice deficient in ORMDL1 alone or with ORMDL2 (O1/2dKO) or ORMDL3 (O1/3dKO). For comparison, we also derived BMMCs from mice deficient in ORMDL2 (O2KO) and ORMDL3 (O3KO). BMMCs from O1/3dKO mice showed a 60% reduction in total ORMDL protein (Fig. 1a). In O1/2dKO BMMCs, total ORMDL protein levels were reduced by only around 30% (Fig. 1a), suggesting that ORMDL3 is predominantly expressed in BMMCs. This is further attested in O3KO cells, which had comparable ORMDL protein levels with O1/3dKO cells (Fig. 1a). Microarray analysis of mRNA expression profiles confirmed that all three ORMDL paralogs are expressed in murine mast cells, albeit the degree of expression of individual ORMDL members varies between different mast cell subsets (Supplementary Fig. S1d with data obtained from^21^).

Major functions of mast cells in allergic diseases and asthma are commonly mediated by the binding of a multivalent antigen to immunoglobulin E (IgE) bound to the high-affinity IgE receptor (FcεRI) located on the surface of mast cells^22^. Antigen-mediated crosslinking of the IgE-FcεRI complexes on the surface of mast cells initiates a signalling cascade that ultimately leads to calcium mobilization, mast cell degranulation, and the synthesis of lipid mediators and cytokines^23^. FcεRI-mediated degranulation and calcium response of O1KO BMMCs following activation with 500 ng/ml and 1000 ng/ml of antigen was similar to the response observed in antigen-activated BMMCs derived from WT, O2KO, O3KO and O1/2dKO cells (Fig. 1b–d). A significant rise in intracellular calcium above that of WT levels was only observed in O1/3dKO BMMCs, yet without a corresponding increase in degranulation (Fig. 1b–d).

Varying expression of ORMDL proteins have been shown to alter Akt kinase and nuclear factor kappa B (NF-κB) signalling pathways^18,19,24,25^. Levels of phosphorylated Akt kinase at serine 473 (p-Akt) following antigen activation were comparable between the various BMMC populations (Fig. 1e). Levels of phosphorylated inhibitor of nuclear factor kappa B (p-iκB) rose uniformly after 2 and 5 min following antigen activation in WT, O1KO, O2KO and O1/2dKO BMMCs (Fig. 1e). O1/3dKO BMMCs exhibited elevated phosphorylation of iκB after 5 min following antigen activation compared with WT cells (Fig. 1e). In O1/3dKO BMMCs, raised p-iκB protein concentrations corresponded with increased relative mRNA levels of tumour necrosis factor alpha (TNF-α) (Fig. 1f). Transcript levels of interleukin (IL)-6 and IL-13 were not upregulated in the various BMMC populations (Fig. 1f). The changes in signalling events and cytokines following antigen activation of O1/3dKO BMMCs mirrored those observed in O2/3dKO cells investigated in our previous study^19^.

### Characterisation of BMMCs with reduced levels of all three ORMDL paralogs

We attempted to generate mice with simultaneous deletions of all three ORMDL proteins to determine functional redundancy and examine potential interchangeable roles between the three mammalian paralogs. However, crossbreeding did not produce any triple ORMDL KO mice, as this genotype is not viable, a finding consistent with previous observations^26^. As such, we reduced the expression of all three ORMDL proteins by knockdown (KD) of *Ormdl3* using short hairpin RNAs (shRNA) in BMMCs derived from O1/2dKO mice (O1/2dKO-O3KD cells). Using this approach, we reduced the levels of all three ORMDL proteins in primary cells to emulate near normal physiological conditions. For comparison purposes, we also prepared WT BMMCs with *Ormdl3* KD (WT-O3KD cells), as well as both WT and O1/2dKO cells transfected with non-target (NT) vectors—WT-NT and O1/2dKO-NT BMMCs.

**Figure 2:**
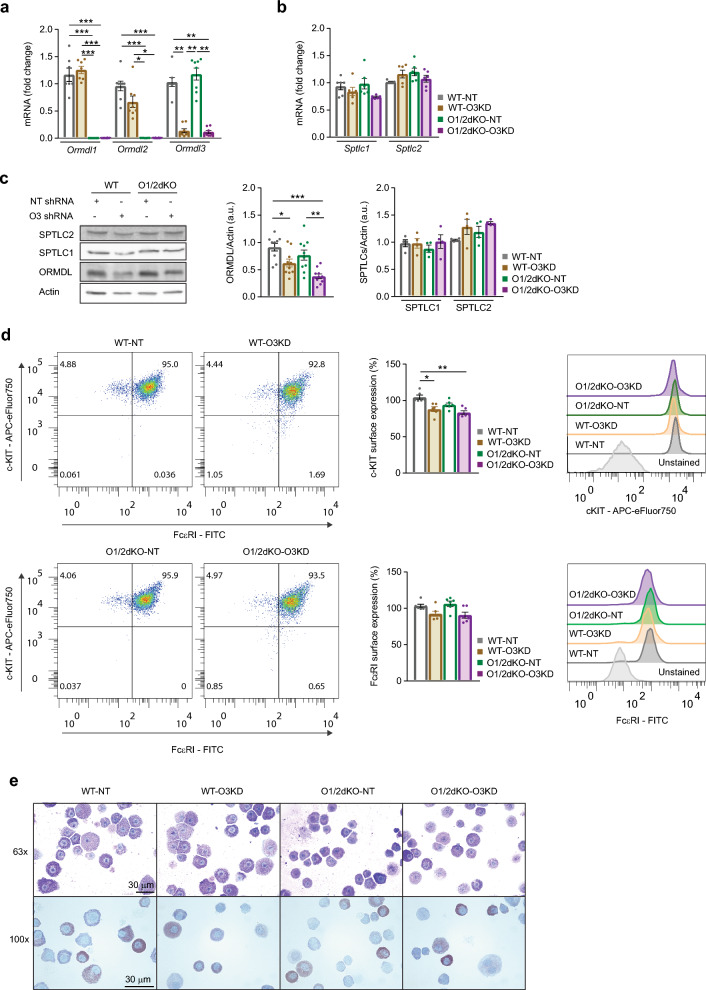
Characterisation and comparison of WT and O1/2dKO BMMCs transfected with NT or O3 KD shRNA. (**a**) Relative mRNA expression of individual *Ormdl* paralogs. n = 8 biological replicates. (**b**) Relative mRNA expression of the two major SPT subunits. n = 6 biological replicates. (**c**) Immunoblot analysis and statistical evaluation of ORMDL proteins and SPT subunits. Actin was used as a loading control. n = 10 and n = 4 biological replicates for ORMDL and SPT subunits, respectively. Uncropped immunoblots are shown in Supplementary Fig. S8. (**d**) Flow cytometric evaluation of surface receptors c-KIT and FcεRI indicating mast cell maturation. n = 6 biological replicates. (**e**) Morphological evaluation of the two transfected cell types using cytological stains. MGG- (top panel) and toluidine blue-stained (bottom panel) cytospin preparations of the BMMCs. Magnification is 63x (top panel) and 100x (bottom panel). Scale bars represent 30 μm. Data were analysed by Kruskal–Wallis test with Dunn’s posttest (**a** and** d**) or one-way ANOVA with Tukey’s posttest (**b** and **c**). *p < 0.05, **p < 0.01 and ***p < 0.001. All the results are represented as mean ± SEM.

At the mRNA level, silencing *Ormdl3* in WT (WT-O3KD) and O1/2dKO (O1/2dKO-O3KD) cells decreased ORMDL3 transcripts by about 80% (Fig. 2a). However, at the protein level, the amount of total ORMDL protein was reduced by only about 40% in WT-O3KD cells and 60% in O1/2dKO-O3KD cells (Fig. 2c). The discrepancy between ORMDL transcript and protein levels are likely due to post-translational regulation and degradation pathways that ultimately determine protein quantities^27,28^. Both mRNA expression and protein levels of the two major SPT enzyme subunits, SPTLC1 and 2, remained unchanged in the transfected cells (Fig. 2b,c). Flow cytometry analysis of FcεRI and c-KIT (CD117), surface receptors of mast cell maturation and differentiation, revealed decreased c-KIT levels in cells with ORMDL3 KD, while FcεRI expression remained unchanged (Fig. 2d). Mast cell morphology of the BMMCs was unaltered by the transfections, as was evident from analysis of the cells stained with May-Grünwald Giemsa (MGG) and toluidine blue, which show characteristic intracellular granules (Fig. 2e). This prompted us to use the transgenic cells to study the effects that decreased ORMDL proteins have on mast cell functions.

### Role of ORMDL proteins in the synthesis of sphingolipids in mast cells

**Figure 3:**
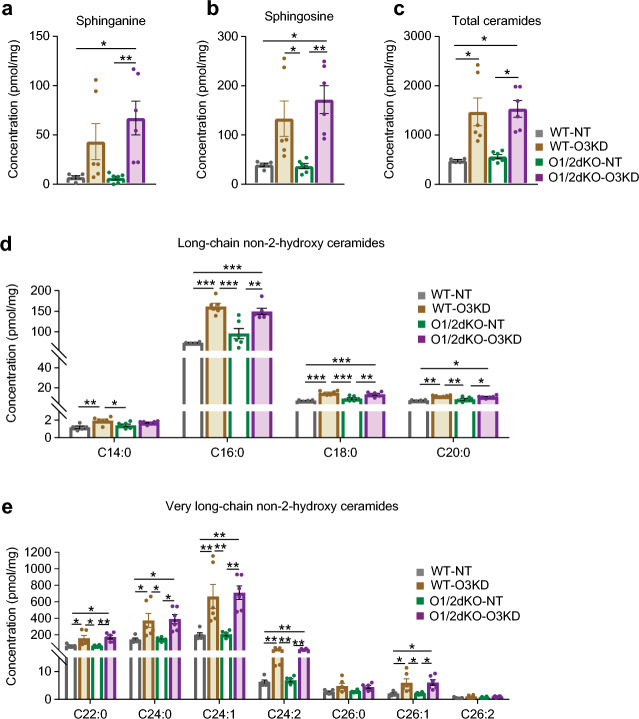
Role of ORMDL proteins in BMMC sphingolipid biosynthesis. Concentrations of (**a**) sphinganine (C18:0), (**b**) sphingosine (C18:1) and (**c**) total ceramides (d18:1 sphingoid base) measured by LC–ESI–MS/MS. Concentrations of the different non-2-hydroxy ceramide molecular species (d18:1 sphingoid base) with (**d**) long-chain and (**e**) very long-chain fatty acid side chains as measured by LC–ESI–MS/MS. Data were analysed by Kruskal–Wallis test with Dunn’s posttest (**a**, **b** and **c**) or one-way ANOVA with Tukey’s posttest (**d**). n = 6 biological replicates for all measurements. *p < 0.05, **p < 0.01 and ***p < 0.001. All the results are represented as mean ± SEM.

As ORMDL proteins are involved in sphingolipid biosynthesis, we decided to assess this role in mast cells. Sphingolipids were extracted from whole BMMCs and analysed by tandem mass spectrometry (LC-ESI-MS/MS). Though the concentration of sphinganine appears to be increased following the lone silencing of *Ormdl3* in WT BMMCs when compared with WT-NT cells, this increase is not statistically significant (Fig. 3a). The concentration of sphinganine is considerably dispersed between the individually measured samples with a coefficient of variance (CV) of 103.03% in WT-O3KD versus 48.70% in WT-NT cells. This variability likely correlates with the degree of ORMDL3 KD attained in each individual cell (Fig. 2a,c), which is subject to the extent of protein synthesis as well as degradation. The inhibition exerted by ORMDL3 on the SPT enzyme was likely maintained in cells with ineffective repression of the ORMDL3 protein, reflecting the lower sphinganine levels measured. A similar trend is observed with concentrations of sphingosine (Fig. 3b), which is produced primarily by the salvage pathway^7^ and indicates higher sphingolipid turnover, likely secondary to increased de novo biosynthesis (Fig. 3a,c–e). The CV of sphingosine concentrations in WT-O3KD cells is 66.64%, while in WT-NT it is 17.70%. Despite variability in the concentrations of intermediary lipids from both the de novo and salvage pathways, the increased sphingolipid synthesis as a result of loss of SPT enzyme inhibition by decreased ORMDL3 levels is reflected in the significant increase in total ceramide levels in WT-O3KD cells (Fig. 3c). The rise in total sphingolipids in WT-O3KD cells is also manifested by the increased concentrations of individual long- and very long-chained ceramide molecular species (Fig. 3d,e).

Interestingly, in O1/2dKO-NT cells, concentrations of both sphinganine and sphingosine remained unchanged when compared with WT-NT cells (Fig. 3a,b). Consequently, no rise in total ceramides nor various ceramide species was observed (Fig. 3c–e). Only additional KD of *Ormdl3* effectively alleviated SPT enzyme inhibition, as is evident from the increase in concentrations of sphingolipids in O1/2dKO-O3KD cells (Fig. 3a–e). Single deletions of *Ormdl1* or *Ormdl2* were similarly insufficient in increasing ceramide levels in BMMCs (Supplementary Fig. S2c–e). It appears that in BMMCs, the presence of ORMDL3 protein alone is sufficient in maintaining inhibition on the SPT enzyme, as we did not detect an increase in sphinganine, sphingosine nor ceramide levels in O1/2dKO cells (Supplementary Fig. S2a–e). However, additional deletion of *Ormdl1* in the presence of *Ormdl3* KO potentiated sphingolipid synthesis, reflected in the rise in concentrations of sphinganine, sphingosine and total ceramides, as well as individual long- and very long-chain ceramide species in O1/3dKO cells (Supplementary Fig. S2a–e). These results indicate that ORMDL1 and ORMDL2 are partially redundant in sphingolipid biosynthesis in BMMCs, which is consistent with our previous findings^19^.

### Effect of reduced expression of ORMDL proteins on mast cell activation

**Figure 4:**
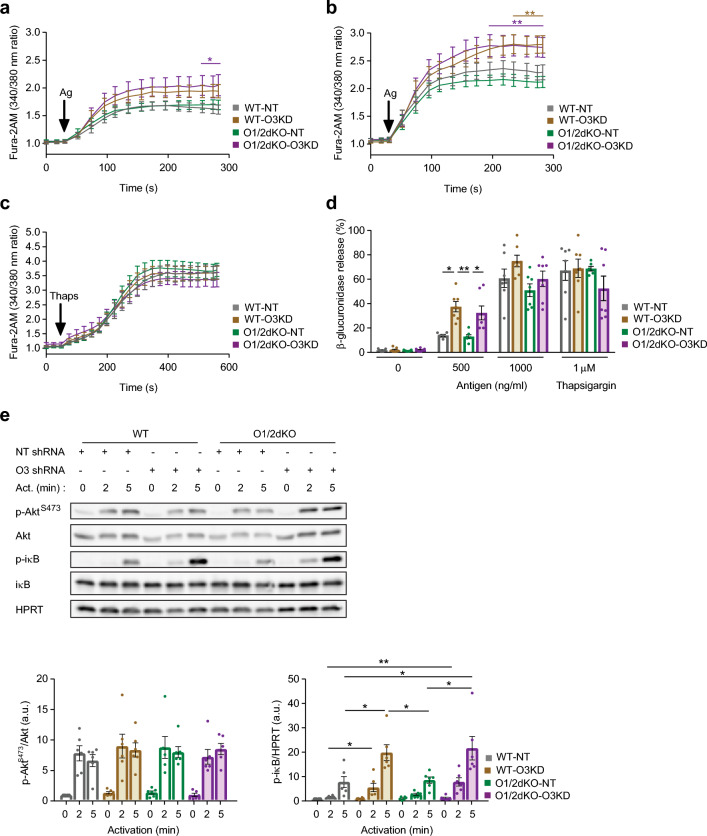
Impact of double ORMDL1 and 2 KO with or without ORMDL3 KD on the activation of BMMCs. Fura-2AM fluorescence as a measure of intracellular calcium at baseline and following activation with (**a**) 500 ng/ml TNP-BSA antigen, (**b**) 1000 ng/ml TNP-BSA antigen and (**c**) 1 µM thapsigargin. Black arrows indicate times at which activators were added to cells. n = 6–8 biological replicates. (**d**) Release of β-glucuronidase as a measure of degranulation in BMMCs activated with 500 ng/ml and 1000 ng/ml TNP-BSA antigen or 1 µM thapsigargin. n = 6–8 biological replicates. (**e**) Immunoblot analysis with statistical evaluation of p-Akt, p-iκB and corresponding loading controls from lysates of non-activated and activated BMMCs. BMMCs were activated with 1000 ng/mL TNP-BSA antigen for 2 and 5 min as indicated. n = 6 biological replicates. Uncropped immunoblots are shown in Supplementary Fig. S9. Data were analysed by two-way ANOVA with Tukey’s posttest (**a**–**c**), one-way ANOVA with Tukey’s posttest (**d**) or Kruskal–Wallis test with Dunn’s posttest (**e**). *p < 0.05 and **p < 0.01. In (**a**), colour of line and asterisk indicates statistical significance of WT-NT versus O1/2dKO-O3KD. In (**b**), colour of lines and asterisks indicate statistical significance of O1/2dKO-NT versus corresponding phenotypes—WT-O3KD = brown and O1/2dKO-O3KD = purple. All the results are represented as mean ± SEM. Ag—Antigen, Thaps—thapsigargin, Act. – Activation.

We further investigated whether ORMDL3’s dominant role is conserved in the context of mast cell functions. Activation of BMMCs using 500 ng/ml of antigen led to an increase in intracellular calcium levels in O1/2dKO-O3KD BMMCs above that of WT-NT cells (Fig. 4a). At higher antigen concentrations of 1000 ng/ml, the differences in intracellular calcium became more pronounced and revealed elevated levels in WT-O3KD cells as well (Fig. 4b). Intracellular calcium levels of O1/2dKO-NT BMMCs were comparable to those of WT-NT cells at both antigen concentrations (Fig. 4a,b). The differences in calcium levels in BMMCs with *Ormdl3* KD appear to be FcεRI-mediated, as the addition of thapsigargin stimulated comparable rises in calcium in the various BMMC populations (Fig. 4c).

In keeping with calcium mobilisation, no differences in degranulation were noted between the various BMMC populations following the addition of thapsigargin (Fig. 4d). Activation of the BMMCs via FcεRI receptors with 500 ng/ml antigen indicated that only WT and O1/2dKO mast cells with additional KD of ORMDL3 had higher degranulation levels (Fig. 4d). Activation with higher concentrations of antigen caused a further rise in the degree of degranulation in the mast cells and any variations between the cells evened out (Fig. 4d). To verify that the degranulation measurements were not influenced by potential disparities in β-glucuronidase composition between the various cells, we measured the surface expression of CD107a before and after mast cell activation as an indicator of degranulation^29^. Activation of BMMCs with antigen or thapsigargin for 30 min did not reveal any differences in CD107a surface expression between the various cells (Supplementary Fig. S3a). However, WT-O3KD and O1/2dKO-O3KD cells expressed more CD107a on their surface following activation with 500 ng/ml and 1000 ng/ml antigen for 15 min despite expressing less CD107a prior to activation (Supplementary Fig. S3b). Thapsigargin caused a uniform rise in CD107a surface expression in the BMMCs after 15 min (Supplementary Fig. S3b). The dynamics of IgE-mediated activation of BMMCs appears to not only be dependent on the concentrations of antigen but also on the duration of activation, in particular when different elements of a response are measured. WT-O3KD and O1/2dKO-O3KD mast cells demonstrated higher sensitivity to antigen, as was evident from β-glucuronidase release, and had a more rapid onset of activation, evident from CD107a surface expression. At higher concentrations of antigen and longer duration of activation, less sensitive WT-NT and O1/2dKO-NT mast cells responded similarly to WT-O3KD and O1/2dKO-O3KD cells.

Similarly to the calcium and degranulation response, compared with WT-NT, activation of FcεRI receptors induced higher phosphorylation of iκB after 2 and 5 min of addition of antigen only in BMMCs with ORMDL3 KD (Fig. 4e). No significant differences in levels of p-iκB were observed between WT-O3KD and O1/2dKO-O3KD cells (Fig. 4e). This suggests that decreased ORMDL3 has the greatest impact on the NF-κB signalling pathway in BMMCs. We did not observe any differences in the phosphorylation of Akt upon antigen stimulation between the various cell populations (Fig. 4e).

**Figure 5:**
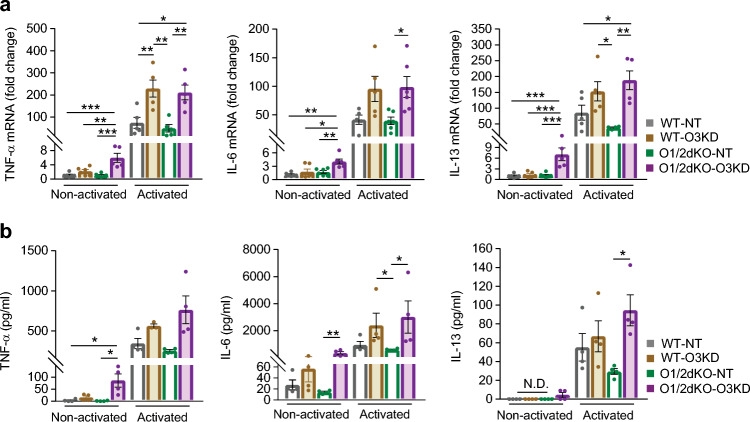
Role of ORMDL proteins in the production of cytokines. (**a**) Relative mRNA expression of TNF-α, IL-6 and IL-13 in non-activated BMMCs and BMMCs activated with 1000 ng/ml TNP-BSA for 1 h. n = 6 biological replicates. (**b**) Concentration of secreted TNF-α, IL-6 and IL-13 cytokines measured in supernatants of non-activated BMMCs and BMMCs activated with 1000 ng/ml TNP-BSA for 5 h. n = 4 biological replicates. Data were analysed by one-way ANOVA with Tukey’s posttest (**a**) or Kruskal–Wallis test with Dunn’s posttest (**b**). *p < 0.05, **p < 0.01 and ***p < 0.001. All the results are represented as mean ± SEM. N.D.—not detected.

To determine whether the abovementioned changes in levels of p-iκB had physiologic consequences, we measured the production of pro-inflammatory cytokines. Consistent with transcriptional regulation by the p-iκB/NF-κB axis, WT-O3KD and O1/2dKO-O3KD exhibited an increased expression of TNF-α compared with WT-NT and O1/2dKO-NT BMMCs following antigen activation (Fig. 5a). In addition to TNF-α, O1/2dKO-O3KD BMMCs also displayed elevated transcript levels of IL-6 and IL-13 (Fig. 5a). Interestingly, even non-activated O1/2dKO-O3KD mast cells, that is cells that were sensitised with IgE but not exposed to antigen, exhibited raised transcript levels of pro-inflammatory cytokines (Fig. 5a). Cells not incubated with IgE, unsensitised BMMCs, exhibited raised expression of IL-13 only when all three ORMDL proteins were reduced (Supplementary Fig. S4). Non-activated O1/2dKO-O3KD mast cells secreted higher amounts of TNF-α and IL-6 cytokines compared with WT-NT and O1/2dKO-NT cells (Fig. 5b). Higher concentrations of secreted IL-6 were likewise detected in supernatants of activated WT-O3KD and O1/2dKO-O3KD cells, while IL-13 was elevated only in O1/2dKO-O3KD cells (Fig. 5b). IL-13 was below the detectable threshold set by the assay in non-activated WT-NT, WT-O3KD and O1/2dKO-NT cells (Fig. 5b). Changes in concentrations of secreted cytokines were not as pronounced between the various cell types as their expression levels (Fig. 5a,b). BMMCs with increased expression of cytokines may have reached their maximal protein synthesis as well as secretory capacity, reflecting the uniform quantities of secreted cytokines despite elevated expression levels.

**Figure 6:**
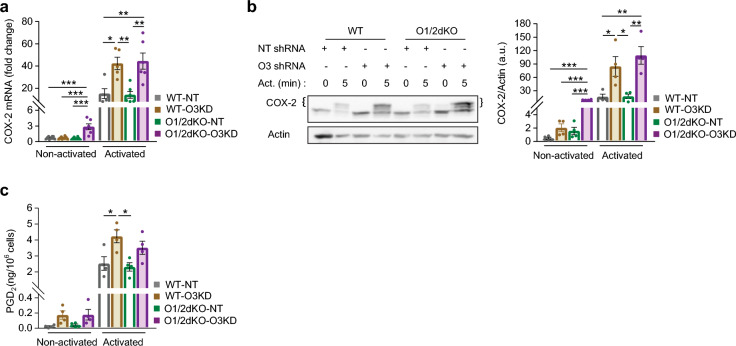
Impact of reduced levels of ORMDL proteins on COX-2 enzyme expression and prostaglandin D_2_ (PGD_2_) production in BMMCs. (**a**) Relative mRNA expression of COX-2 enzyme in non-activated BMMCs and BMMCs activated with 1000 ng/ml TNP-BSA antigen for 1 h. n = 6 biological replicates. (**b**) Immunoblot analysis with statistical evaluation of COX-2 levels from lysates of non-activated BMMCs and BMMCs activated with 1000 ng/mL TNP-BSA antigen for 5 h. n = 4 biological replicates. Curly brackets indicate COX-2 protein bands (upper bands). Actin was used as a loading control, except for one membrane, where β-tubulin was used instead. Uncropped immunoblots, including membrane with β-tubulin as loading control, are shown in Supplementary Fig. S10. (**c**) PGD_2_ production in non-activated BMMCs and BMMCs activated with 1000 ng/ml TNP-BSA antigen for 5 h. PGD_2_ was measured in cell-free supernatants by immunoassay. n = 4 biological replicates. Data were analysed by one-way ANOVA with Tukey’s posttest. *p < 0.05, **p < 0.01 and ***p < 0.001. All the results are represented as mean ± SEM. Act.—Activation.

Activated mast cells with decreased levels of ORMDL3, WT-O3KD and O1/2dKO-O3KD cells, also exhibited elevated expression of cyclooxygenase-2 (COX-2) enzyme compared with WT-NT and O1/2dKO-NT cells (Fig. 6a). Western blotting confirmed that WT-O3KD and O1/2dKO-O3KD BMMCs had a raised expression of COX-2 enzyme at the protein level (Fig. 6b). In keeping with the pattern of cytokine production, sensitised O1/2dKO-O3KD BMMCs exhibited increased expression of COX-2 even in the absence of antigen activation (Fig. 6a,b). Expression of COX-2 remained unchanged in unsensitised cells (Supplementary Fig. S4). COX-2 is an inducible enzyme that catalysis the conversion of arachidonic acid to pro-inflammatory lipid mediators known as prostanoids^30,31^. To investigate the physiologic effects of elevated COX-2 expression, we measured the secretion of prostaglandin D_2_ (PGD_2_) in cell-free supernatants of non-activated and antigen-activated BMMCs. PGD_2_ is a stable downstream product of COX-2 that is produced in abundance by activated mast cells^32^. PGD_2_ production was elevated in WT-O3KD cells following antigen activation (Fig. 6c). Despite elevated COX-2 expression at both the mRNA and protein level, PGD_2_ concentrations in supernatants from non-activated and antigen-activated O1/2dKO-O3KD mast cells remained statistically comparable to those of WT-NT cells (Fig. 6c). PGD_2_ synthesis and secretion involves multiple steps, which involve other enzymes in addition to COX-2^31^. O1/2dKO-O3KD mast cells have a lower threshold for inducing COX-2 expression even in a steady state, yet COX-2 does not appear to be the limiting factor dictating the production of the final lipid mediator.

Altogether, our findings indicate that mast cells with lone or concurrent deletions of ORMDL1 and 2 proteins do not vary phenotypically from WT cells. However, reducing the levels of ORMDL3 in differentiated mast cells causes a pro-inflammatory phenotype, which is further potentiated by the loss of ORMDL1 and 2, as is evident from increased pro-inflammatory mediators in O1/2dKO-O3KD BMMCs even at a resting state in the absence of antigenic stimuli.

## Discussion

Multiple studies have linked ORMDL proteins, in particular ORMDL3, with inflammatory diseases^33^ in which mast cells play important roles^1,2^. In the current study, we generated O1KO mice and subsequently, via crossbreeding, O1/2dKO mice. By isolating BMMCs from O1/2dKO mice and silencing *Ormdl3* in them, we were able to study the effect of reducing the levels of all three ORMDL proteins simultaneously on mast cell physiology.

The highly homologous nature of the ORMDL family of proteins suggests that the individual proteins are, at least partially, capable of substituting each other. As such, complete deletions of individual protein members during embryonic development likely lead to compensatory mechanisms that ensure homeostatic regulation of vital functions. Effective compensatory mechanisms safeguard against severe abnormalities and assure the survival of an organism. This concept is reflected in our current and previous work^19^, in which we generated viable mice with single and double deletions of ORMDL proteins. Though capable of partially compensating for each other, the transgenic mice illustrate that the individual members of the ORMDL family of proteins are not equally indispensable and display functional redundancy. As we demonstrated, mice with simultaneous deletions of ORMDL1 and 3 vary significantly in stature from WT and other ORMDL double, as well as single, KO mice. In addition, the absence of both ORMDL1 and 3 leads to neurodegeneration and lower mouse viability, which is not evident in mice with sole ORMDL3 deletions nor ORMDL2 and 3 double KO^26^. Our observations and those made by another research group^26^, indicate that complete deletion of all three ORMDL proteins is incompatible with life. To date, only one study described the simultaneous KO of all three ORMDLs, but this was done under in vitro conditions in an immortalised cell line^34^. In other studies, ORMDL proteins were knocked down using siRNAs, maintaining partial expression of the proteins^13,14,18,35,36^.

Among immune cells, mature mast cells are relatively long-lived. BMMCs can be grown and are functional in vitro for 2 to 5 months^37^. We exploited these properties to prepare primary immune cells with reduced expression of all three ORMDL proteins by silencing *Ormdl3* in O1/2dKO BMMCs using shRNA. As shown in the results section, levels of mRNA transcripts are an insufficient indicator to ascertain the degree of decrement of ORMDL proteins attained in cells. At the mRNA level, expression of ORMDL proteins appeared almost completely suppressed in O1/2dKO-O3KD cells, with no expression of ORMDL1 and 2, and more than a five-fold reduction in ORMDL3 expression. However, at the protein level, we noted fluctuations in ORMDL protein expression ranging from only two to about a four-fold decrease compared to WT-NT cells. Quantitative evaluation of specific ORMDLs at the protein level is complicated by cross-reactivity of antibodies directed against individual ORMDL paralogs due to the ORMDL proteins’ high sequence homology. In our study, variability of ORMDLs at the protein level in O1/2dKO-O3KD cells can be assumed to correspond to levels of ORMDL3 as both ORMDL1 and 2 have been knocked out. The observed variability in ORMDL3’s expression in O1/2dKO-O3KD cells and differences between mRNA and protein levels are likely a reflection of ORMDL3’s complex post-translational regulation coupled with its degradation at the protein level.

Our results indicate that sphingolipid biosynthesis is closely dependent on the amount of ORMDL3 present in mast cells. In BMMCs with single ORMDL KOs, only the deletion of ORMDL3 increased sphingolipid production. Absence of ORMDL1 or 2 alone had no effect on sphingolipid levels. This is consistent with previous research, which showed that only cells with deleted or downregulated ORMDL3, but not ORMDL1 or 2, exhibited changes in sphingolipid production^19,26,34–36^. In some cell types, even the reduction of ORMDL3 was insufficient in increasing sphingolipid production and the reduction of all three ORMDL proteins was necessary to alter sphingolipid levels^14^. In our study, ORMDL3 deletion caused a one to two-fold increase in sphingolipid concentrations, while the simultaneous deletion of ORMDL1 with ORMDL3 increased the production further to about three-fold. Concurrent deletion of ORMDL1 with ORMDL2 had no effect on sphingolipid synthesis. In our previous study, we noted that the deletion of ORMDL2 together with ORMDL3 also caused an increase in sphingolipid levels, which were three to five-fold higher compared with WT cells^19^. Altogether, our findings indicate that de novo sphingolipid synthesis in mast cells is predominantly regulated by ORMDL3, while ORMDL1 and 2 appear to be functionally redundant. However, in the absence of ORMDL3, additional deletion of ORMDL1 or 2 potentiates the increased sphingolipid production. The dominancy of ORMDL3 in the regulation of de novo sphingolipid biosynthesis is further reflected in WT-O3KD and O1/2dKO-O3KD mast cells, where fluctuations in ORMDL3 levels dictate the degree of inhibition exerted on the SPT enzyme as is demonstrated by the relatively high coefficients of variation in sphingolipid measurements in these cells.

As previously mentioned, in our current and previous study^19^, we noted that either the absence of ORMDL1 or 2 potentiates sphingolipid synthesis in mast cells during the concurrent absence of ORMDL3. Interestingly, in murine brain tissue, only additional deletion of ORMDL1 was able to further increase sphingolipid production above the concentrations observed in O3KO mice. Sphingolipid levels from brain tissues of O2/3dKO mice were identical to levels measured in WT mice^26^. It therefore appears that interactions between the individual ORMDL proteins and the SPT enzyme are cell and tissue specific, depending on the degree of expression of each individual ORMDL paralog. The discovery of the detailed structure of the SPT-ORMDL3 complex indicates that the overall lipid environment affects the conformation of ORMDL3 in the complex^38^. Furthermore, ORMDL proteins are not the only known regulators of de novo sphingolipid synthesis. The ER membrane protein Nogo-B has also been identified as an inhibitor of the SPT enzyme^39^. Different lipid environments and expression levels of other regulators of the SPT enzyme may account for the varying sphingolipid concentrations observed between distinct cell types.

With regard to the role of individual ORMDL proteins in mast cell signalling, O1KO mast cells, similarly to previously described O2KO mast cells^19^, showed no changes in the measured parameters, which included intracellular calcium concentrations, degranulation, phosphorylation of iκB, and expression of pro-inflammatory cytokines following activation via FcεRI receptors. Antigen-activated O1/3dKO mast cells exhibited increased intracellular calcium concentrations and p-iκB levels. Similar changes were observed in O2/3dKO mast cells in our earlier study^19^. We previously reported that downregulation of ORMDL3 in mast cells leads to an increase in pro-inflammatory responses via Akt and NF-κB signalling pathways^18^. In our current study, we did not observe any changes in p-Akt levels between the various BMMC populations. This may be explained by the different mice strains used in the studies, which exhibit certain phenotypic dissimilarities based on their genotypic background^40^.

The nature of mast cell responses to antigenic stimuli are not solely governed by their genotypic background but also by their microenvironment. The microenvironment that maturing and differentiating mast cells grow in influences the expression of genes and subsequent physiology^21^. In our previous study, we noted a rise in intracellular calcium concentrations in O3KO BMMCs above those measured in WT cells^19^, a finding, which was not reproduced in the present study. The inconsistencies in the results may be due to slight variations in growth conditions. While in our current study BMMCs were grown in the presence of commercially available, recombinant stem cell factor (SCF) and IL-3, BMMCs in our earlier study were grown using 4% of culture supernatants from cells that produce SCF and IL-3. Supernatants from cultured cells contain many biologically active molecules that may influence the reactivity of BMMCs^41^.

Consistent with our previous findings^18,19^, we noted that mast cells, which exhibited elevated activation of the NF-κB signalling pathway, as measured by increased phosphorylation of iκB, had raised levels of pro-inflammatory cytokines. In O1/3dKO BMMCs, increased p-iκB was accompanied by upregulated TNF-α expression. Several inconsistencies in IgE-mediated responses were noted between O3KO and WT-O3KD BMMCs. Antigen-activated WT-O3KD BMMCs demonstrated raised calcium concentrations, degranulation and p-iκB levels with upregulated expression of pro-inflammatory cytokines. These findings were not observed in O3KO cells. The apparent increased reactivity of WT-O3KD BMMCs following antigen activation, which was not detected in O3KO cells may be due to homeostatic adaptations that occur at the level of the whole organism following disruptions to the genome. O3KO BMMCs were derived from mice, which carried the genetic mutation from birth. This could have led differentiating and maturing cells to develop adaptive mechanisms to promote survival. As the three ORMDL paralogs are highly homologous, its members may compensate for the loss of one protein during development. In WT-O3KD BMMCs, the mutation was introduced following maturation, thereby making it more difficult for the other two remaining ORMDL proteins to compensate for an acute genomic insult, which manifests as differences in physiological responses. Alternatively, increased reactivity of BMMCs may be dependent on partial ORMDL3 protein expression, which dysregulates normal mast cell physiology.

IgE-sensitised BMMCs with reduced levels of all three ORMDL proteins, O1/2dKO-O3KD cells, expressed increased pro-inflammatory cytokines and COX-2 even at rest in the absence of antigen activation. Several monoclonal IgE antibodies have been described as cytokinergic^42^. Binding of these antibodies causes aggregation of FcεRI receptors even in the absence of antigen^42,43^. This phenomenon was observed with IgE concentrations of 5 μg/mL or higher^42^. In our studies, we used 1 μg/mL of IgE for the sensitisation of BMMCs. As WT-NT BMMCs did not display increased reactivity at rest and unsensitised O1/2dKO-O3KD cells exhibited raised IL-13 transcript levels, the observed pro-inflammatory phenotype of O1/2dKO-O3KD mast cells was likely not due to cytokinergic effects of IgE. However, reduced levels of ORMDL proteins appear to prime mast cells, leading to increased reactivity, which is IgE-dependent.

Sphingolipids, in particular ceramides, affect membrane fluidity and, consequently, influence cell signalling^9^. Elevated ceramide concentrations may affect the organisation of the plasma membrane and lead to the formation of membrane domains, which may promote the crosslinking of FcεRI receptors, leading to mast cell priming. BMMCs derived from O2/3dKO mice had raised levels of sphingosine-1-phosphate (S1P) compared with WT BMMCs^19^. S1P is formed following the phosphorylation of sphingosine by sphingosine kinases and is degraded by S1P lyase^7,8^. As the concentration of sphingosine in O1/2dKO-O3KD cells was elevated, it is likely that S1P is also raised in these cells. BMMCs derived from S1P lyase-deficient mice, which have chronically elevated S1P levels, displayed a hyper-responsive phenotype characterised by enhanced calcium influx, degranulation response and production of pro-inflammatory cytokines TNF-α and IL-6^44^. S1P may contribute to the pro-inflammatory phenotype observed in mast cells with reduced levels of all three ORMDL proteins, as it is also known to prime mast cells and decrease their activation threshold^4^.

As an ER-resident protein, several studies have suggested that ORMDL3 is involved in the regulation of the unfolded protein response (UPR), which is activated as a result of ER stress^45,46^. Overexpression of ORMDL3 in rat basophilic leukaemia (RBL-2H3) cells caused an increase in one of the ER stress sensors, namely protein kinase RNA-like ER kinase (PERK)^47^. In another study, overexpression of ORMDL3 in MC/9 mouse mast cell line upregulated the activating transcription factor 6 (ATF6) ER stress sensor, dampening immune responses^48^. In this study, we did not explore ER stress and the various UPR pathways. However, changes in cellular lipids and, in particular, the composition of ER membrane lipids are also known to activate UPR responses independently of proteotoxic stress^49,50^. The observed phenotypes described in the aforementioned studies may be a consequence of changes in sphingolipid levels that we noted in our current study. Furthermore, the involvement of ORMDL proteins in inflammatory processes likely involves an interplay between multiple metabolic and signalling pathways. Our previous study, in which we described the interactions between ORMDL3, SPT and 5-lipoxygenase enzymes^20^, highlights the concept of multiple interconnected functional pathways.

Overall, our results show that ORMDL3 is the dominant member of the ORMDL family of proteins in regulating mast cell functions and homeostasis. Reduction of ORMDL3 protein levels increases the expression of pro-inflammatory cytokines and COX-2. This phenomenon is further enhanced by the loss of ORMDL1 and 2, such that mast cells with reduced levels of all three ORMDL proteins have a decreased threshold for activation and produce pro-inflammatory mediators even in the absence of a specific antigen-mediated stimulus.

## Methods

### Mice

The generation of ORMDL2 and ORMDL3 KO mice was described previously^19^. ORMDL1 KO mice were generated using CRISPR/Cas9 genome editing. For this purpose, Cas9 mRNA was combined with two single-guide RNAs (sgRNAs) targeting the second exon in the *Ormdl1* gene prior to microinjection into fertilised zygotes. sgRNAs were synthesised using a MEGAshortscript T7 kit (Invitrogen, AM1354) according to the manufacturer’s instructions to generate the following sequences: sgRNA_*Ormdl1* forward: 5′-ACTCGTGTAATGAACAGCCG-3′ and sgRNA_*Ormdl1* reverse: 5′-TTGGTTAAGGTCCAGGCAAC-3′. Genome editing was confirmed by PCR-based heteroduplex mobility shift assay^19^ using the following primers specific for *Ormdl1*—forward: 5′-GTCTACCTAACAGTAGTGACAG-3′ and reverse: 5′-GAACCACCAGTAACTTATCTCC-3′. Offspring from the first filial generation harboring an 85 base pair deletion within the second exon leading to a frameshift mutation in *Ormdl1* were backcrossed six times into the C57BL/6 J genetic background and used for further breeding. O1/2dKO and O1/3dKO mice were generated by crossbreeding of established ORMDL1, ORMDL2 and ORMDL3 homozygous mutants. Genotyping of progeny was performed by end-point PCR using DNA isolated from tail-snip. Primers used for end-point PCR for *Ormdl* genes are listed in Supplementary Table S3. PCR products were size-fractioned by electrophoresis on 5% Tris base, boric acid and EDTA polyacrylamide gels (TBE-PAGE) to identify frameshift mutations. All mice were housed in top-filter cages and fed a standard diet with freely available water and food. All work with animals was conducted in accordance with the Institute of Molecular Genetics guidelines (permit number 12135/2010-17210) and national guidelines (2048/2004-1020). All experiments performed using mice were approved by the ethical committee of the Institute of Molecular Genetics and were conducted in accordance with the ARRIVE guidelines. For the purposes of this study, age- and sex-matched mice were used for the isolation of bone marrow cells as described below.

### Cells and lentiviral infection

Bone marrow cells were isolated from femurs and tibias of 4 to 7 week-old WT and transgenic mice. The isolated cells were cultured to obtain bone marrow-derived mast cells (BMMCs) in RPMI 1640 medium (Sigma, Cat. No. R8758) supplemented with antibiotics (100 U/ml penicillin, 100 μg/ml streptomycin, Gibco), 71 μM 2-mercaptoethanol, minimal essential medium non-essential amino acids, 0.7 mM sodium pyruvate, 2.5 mM L-glutamine, 12 mM D-glucose, 10% fetal calf serum (FCS; Biosera, Cat. No. FB-1090/500), 15 ng/ml recombinant murine SCF (PeproTech EC, Cat. No. 250-03) and 20 ng/ml recombinant murine IL-3 (PeproTech EC, Cat. No. 213-13). Cells with *Ormdl3*-specific KD and corresponding controls were prepared by lentiviral transduction of 8 week-old mature BMMCs using HEK 293T/17 packaging cells for preparation of virus. Suitable short hairpin RNAs cloned into pLKO.1 vector were purchased from Open Biosystems. Equimolar mixes of TRCN0000126200 and TRCN0000126203 were used for preparation of *Ormdl3* KD. Non-target pLKO.1 vector was used as a negative control^18^.

### Cell surface receptors

BMMCs were tested for the presence of c-KIT (CD117) and FcεRI on the cell surface 8 weeks after cultivation and used for experiments for a further 2 to 3 months. BMMCs (0.5 × 10^6^) from culture were centrifuged (500×*g*, 5 min), washed with phosphate-buffered saline (PBS) and stained with 1 μg/ml FITC-conjugated anti-FcεRI (clone MAR-01, eBioScience) and APC-eFluor 780-conjugated anti-CD117 (clone 2B8, eBioScience) in PBS supplemented with 2% FCS for 30 min in the dark on ice. Fluorescence was measured using a BD Symphony flow cytometer.

### Cell sensitisation and activation

BMMCs were cultured for 16 h in SCF- and IL-3-free RPMI 1640 medium supplemented with 10% FCS and 1 μg/mL 2, 4, 6-trinitrophenol (TNP)-specific mouse IgE (IGEL b4 1^51^). The cells were then washed with buffered saline solution (BSS; 20 mM HEPES, pH 7.4, 135 mM NaCl, 5 mM KCl, 1.8 mM CaCl_2_, 5.6 mM glucose, 1 mM MgCl_2_) supplemented with 0.1% bovine serum albumin (BSA) and activated with 500 ng/ml or 1000 ng/ml antigen (TNP-BSA conjugate, 15–25 mol TNP/mol BSA) or 1 µM thapsigargin (Invitrogen, Cat. No. T7459) at 37 °C for the indicated times.

### Calcium response

Changes in free intracellular calcium concentrations were measured as described previously^19^. Briefly, sensitised BMMCs (0.2 × 10^6^) were loaded with 1 ng/mL Fura-2AM in the presence of 2.5 mM probenecid for 30 min at 37 °C under gentle agitation. Thereafter, the cells were washed and incubated with probenecid for a further 15 min. After the incubation, the cells were washed in BSS-0.1% BSA and aliquoted into a white 96-well plate. Intracellular concentrations of calcium before and after cell activation with the indicated concentrations of antigen or thapsigargin were measured by spectrofluorometry at excitation wavelengths of 340 nm and 380 nm, with a constant emission wavelength of 510 nm using an Infinite 200 M plate reader (TECAN).

### Degranulation: β-glucuronidase assay

The percentage of released β-glucuronidase from BMMCs was used as one measure of the degree of cellular degranulation^52^. Sensitised BMMCs (0.2 × 10^6^) were activated with the indicated concentrations of antigen or thapsigargin for 30 min. The cells were then centrifuged and 30 μL of supernatants were removed. The supernatants were incubated with 30 μL of 40 μM fluorogenic β-glucuronidase substrate (4-methylumbelliferyl-β-D-glucuronide hydrate) for 60 min at 37 °C under gentle agitation. After 60 min, the reaction was stopped by adding 0.2 M glycine buffer, pH 10.0, and fluorescence was measured using an Infinite 200 M plate reader (TECAN) at excitation and emission wavelengths of 355 nm and 460 nm, respectively. Percentage degranulation was calculated as the ratio of measured fluorescence compared to fluorescence from supernatants of cells that were lysed with 0.5% Triton X-100.

### Degranulation: CD107a surface marker

The expression of CD107a on the surface of BMMCs was used as a second measure of cellular degranulation^29^. Sensitised BMMCs (0.2 × 10^6^) were activated with the indicated concentrations of antigen or thapsigargin for 15 or 30 min. The cells were thereafter centrifuged and stained with 1 μg/ml BD Horizon V450-conjugated anti-CD107a (clone 1D4B, BD Biosciences) in PBS supplemented with 2% FCS for 30 min in the dark on ice. Fluorescence was measured using a BD Symphony flow cytometer.

### Gel electrophoresis and immunoblotting

Sensitised BMMCs (10^6^) were activated with 1000 ng/ml antigen for the indicated times. Lysates from whole cells were prepared by solubilising antigen-activated and non-activated BMMCs in 30 μL Laemmli sample buffer^53^, sonicating and boiling for 5 min. Proteins from lysates were size-fractioned on 10% or 13.5% sodium dodecyl sulfate–polyacrylamide gels by electrophoresis (SDS-PAGE) and transferred onto nitrocellulose membranes (0.45 μm; GE Healthcare Life Sciences). The membranes were blocked in Tris-buffered saline with Tween 20 (TBST; 20 mM Tris–HCl, pH 7.5, 200 mM NaCl, 0.1% Tween 20) supplemented with 2% BSA and cut into strips corresponding to different molecular weights based on the protein marker used. The membrane strips were probed with the appropriate primary antibodies listed in Supplementary Table S1 for 60 min at room temperature (RT). Non-phosphorylated proteins were visualised on the same membrane as their phosphorylated counterparts following stripping. Bound antibodies were detected by incubating the membranes with the appropriate horseradish peroxide (HRP)-conjugated secondary antibody listed in Supplementary Table S2 for 60 min at RT. The membranes were then washed three times with TBST and visualised with an enhanced chemiluminescence solution^54^ on Luminescent Image Analyser LAS 3000 (FujiFilm). Signal quantification was performed using AIDA software (Raytest, GmbH). The highest exposure of each protein band prior to overexposure was used for signal quantification. Amount of phosphorylated proteins was normalised to corresponding non-phosphorylated loading controls, except for p-ikB, where HPRT was used as a loading control as ikB proteins degrade upon stimulation^55^.

### Quantitative real-time PCR

RNA was isolated from whole cells using MicroElute Total RNA kit (OMEGA bio-tek). M-MLV reverse transcriptase (Invitrogen, Cat. No. 28025013) was used according to the manufacturer’s instructions to produce single-stranded complementary DNA (cDNA). Real-time PCR amplification was performed in 10 μl reaction volumes in 384-well plates using LighCycler 480 II apparatus (Roche Diagnostics) as described previously^18^. Hypoxanthine–guanine phosphoribosyltransferase (HPRT), ubiquitin (UBB) and TATA-binding protein (TBP) were used as reference genes. The expression levels of mRNAs were normalised to the geometric means of the reference genes. All primers used are listed in Supplementary Table S3. Cytokine expression was measured in sensitised BMMCs (2 × 10^6^) following 60 min activation with 1000 ng/ml antigen, while COX-2 was measured after 5 h antigen activation. Alternatively, both COX-2 and cytokines were measured in BMMCs that were not exposed to IgE (unsensitised cells).

### Sphingolipid measurements

The extraction of sphingolipids from BMMCs and analysis by tandem mass spectrometry (LC-ESI-MS/MS) was performed as described previously^20^. BMMCs (6 × 10^6^) were sonicated and protein concentrations were measured using a Pierce BCA protein assay kit according to the manufacturer’s instructions. Aliquots of 40 µg of proteins were transferred into glass vials containing internal standards (C17:1 sphingosine [Cat. No. 860640P] and C17:0, d18:1 ceramide [Cat. No. 860517P], Avanti Polar Lipids) and mixed with 1 ml of 2:1 (v/v) chloroform/methanol solution. The samples were incubated for 60 min at room temperature under gentle agitation and filtered through Millex LH 0.45 µm filters (Merck Millipore) prior to LC–ESI–MS/MS analysis.

### Cytokine immunoassay

Sensitised BMMCs (10^6^) were activated with 1000 ng/ml antigen in 100 µL BSS supplemented with 0.1% BSA for 5 h. Concentrations of secreted cytokines were measured from cell-free supernatants of activated and non-activated BMMCs using a bead-based immunoassay (LEGENDplex Mouse B Effector Panel; BioLegend, Cat No. 740820) according to the manufacturer’s instructions. Data were acquired using a BD Symphony flow cytometer equipped with 488 nm and 637 nm lasers. The results were analysed using LEGENDplex data analysis software.

### PGD_2_ production

Sensitised BMMCs (10^6^) were activated with 1000 ng/ml antigen in 100 µL BSS supplemented with 0.1% BSA for 5 h. The cells were then centrifuged and PGD_2_ concentrations were measured in cell-free supernatants using a competitive enzyme immunoassay according to the manufacturer’s instructions (Prostaglandin D_2_ ELISA Kit; Cayman Chemicals, Cat. No. 512031).

### Morphological analysis

To ensure that the transfection of the WT and O1/2dKO BMMCs did not change the cells’ characteristic mast cell phenotype, the cells’ morphologies were examined under the microscope using May-Grünwald Giemsa (MGG) and toluidine blue cytological stains. Cytospin preparations of the cells (3 × 10^4^) were fixed with ice-cold methanol for 3 min, allowed to air dry and stained with May-Grünwald solution for 5 min. After 5 min, the preparations were stained with Giemsa-Romanowski solution for 10 min. The slides were washed in distilled water, air dried and mounted using DPX mountant. For toluidine blue staining, cytospins were fixed as before and stained with 0.1% toluidine blue dissolved in 0.6 M hydrochloric acid for 5 min. The slides were washed and mounted using 90% glycerol. Images were acquired using Leica DM6000 widefield microscope using a 63×/1.40 oil-immersion objective for MGG samples and 100×/1.40 oil-immersion objective for toluidine blue preparations.

### Statistical analysis

Statistical analysis was performed using GraphPad Prism software version 7.3. Data are presented as mean ± standard error of the mean (SEM) of at least three independent experiments. Comparison of more than two groups was evaluated using one-way analysis of variance (ANOVA) with Tukey’s posttest for normally distributed data or Kruskal–Wallis test with Dunn’s posttest for non-parametric data. Data was tested for normality using the Shapiro–Wilk normality test. Two-way ANOVA with Tukey’s posttest was used for the comparison of two parameters. Statistical outliers were determined using the ROUT method with Q set to 1%^56^. *P* values of less than 0.05 were considered significant.

## Supplementary Information

Supplementary Information.

## Data Availability

All data generated or analysed during this study are included in this published article and its Supplementary Information files.
