# Supplementary material for: Simultaneous reduction of all ORMDL proteins decreases the threshold of mast cell activation
Source: Sci Rep. 2023 Jun 14;13:9615. doi: 10.1038/s41598-023-36344-5 (PMC10267218; doi:10.1038/s41598-023-36344-5)
Supplement: Supplementary file 1 — Supplementary Information. [file 41598_2023_36344_MOESM1_ESM.pdf]

Supplementary Information

Simultaneous reduction of all ORMDL proteins decreases the threshold of mast cell activation

Livia Demkova[1], Viktor Bugajev[1], Pavol Utekal[1], Ladislav Kuchar[2], Björn Schuster[3, 4], Petr Draber[1*], and Ivana Halova[1*]

[1]Laboratory of Signal Transduction, Institute of Molecular Genetics of the Czech Academy of Sciences, Prague, Czech Republic

[2]Research Unit for Rare Diseases, Department of Paediatrics and Inherited Metabolic Disorders, First Faculty of Medicine, Charles University and General University Hospital in Prague, Prague, Czech Republic

[3]Czech Centre for Phenogenomics, Institute of Molecular Genetics of the Czech Academy of Sciences, Prague, Czech Republic

[4]CZ-OPENSCREEN, Institute of Molecular Genetics of the Czech Academy of Sciences, Prague, Czech Republic

*Address correspondence to: Petr Draber or Ivana Halova, Laboratory of Signal Transduction, Institute of Molecular Genetics, Academy of Sciences of the Czech Republic, Videnska 1083, CZ-14220 Prague 4, Czech Republic, Tel.: +420-241 062 468; Fax: +420-241 062 214; E-mail: petr.draber@img.cas.cz, ivana.halova@img.cas.cz

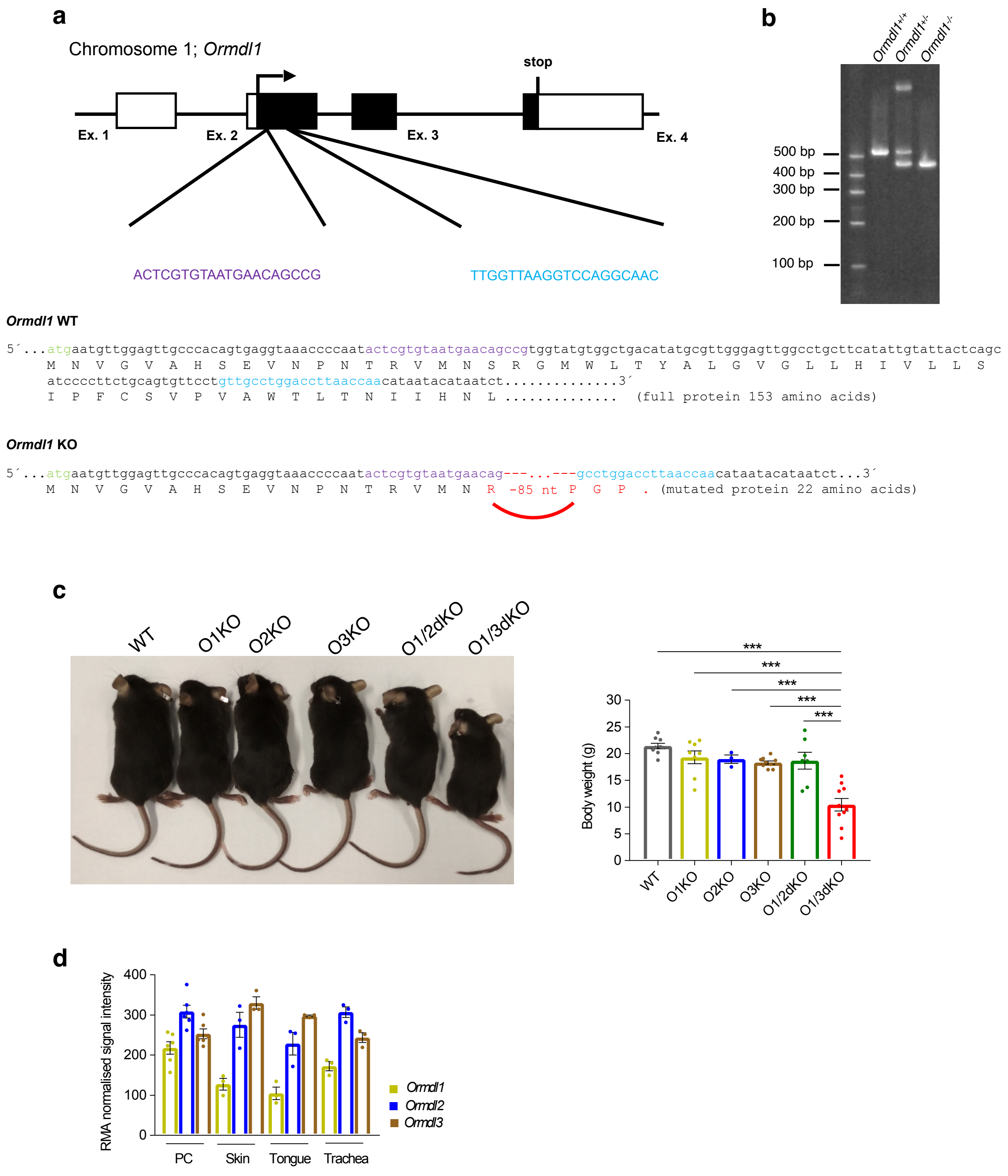

Supplementary Figure S1. Preparation of ORMDL1 KO mice by CRISPR-Cas9 gene editing. (**a**) Schema illustrating the method for the generation of *Ormdl1* KO via CRISPR-Cas9 gene editing. Below, partial DNA and amino acid sequences of ORMDL1 WT and KO proteins indicating the locations of sgRNAs and frameshift mutation in the mutated protein. (**b**) Representative image of TBE-PAGE illustrating the various *Ormdl1* genotypes with notable truncation of the KO mutant gene. (**c**) Image and body weights of 6 week-old female mice with single or double deletions of *Ormdl* genes and corresponding WT mouse. n = 3 – 10 mice per genotype. (**d**) Gene expression levels of the three ORMDL paralogs in different murine mast cell subsets as determined by Affymetrix microarrays (GEO: GSE377448). Data were analysed by one-way ANOVA with Tukey's posttest. *** $p < 0.001$. All the results are represented as mean ± SEM. bp = base pair, nt = nucleotide, RMA = Robust Multiarray Average, PC = peritoneal cavity.

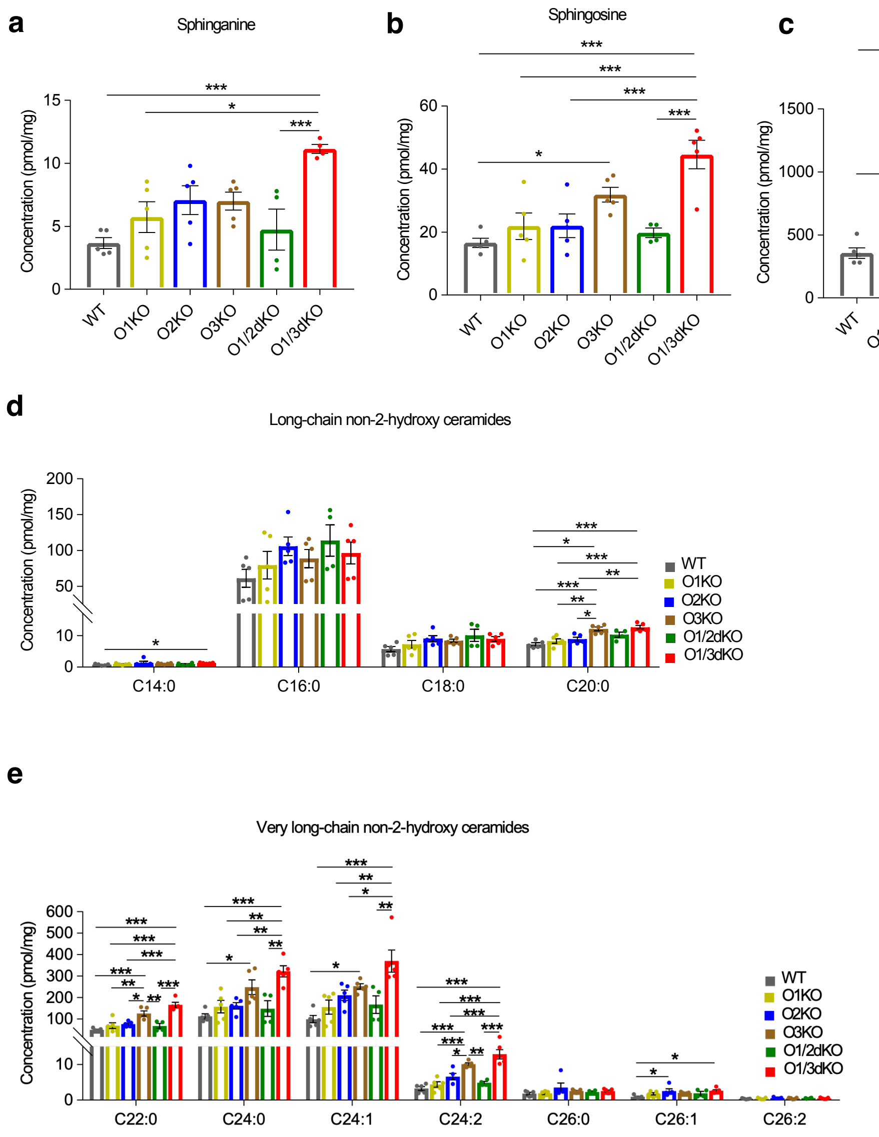

Supplementary Figure S2. Role of the three various ORMDL paralogs in BMMC sphingolipid biosynthesis as indicated by the production of (**a**) sphinganine (C18:0), (**b**) sphingosine (C18:1) and (**c**) total ceramides (d18:1 sphingoid base) measured by LC-ESI-MS/MS. Concentrations of the different non-2-hydroxy ceramide molecular species (d18:1 sphingoid base) with (**d**) long-chain and (**e**) very long-chain fatty acid side chains as measured by LC-ESI-MS/MS. Data were analysed by one-way ANOVA with Tukey's posttest. n = 4 – 6 biological replicates for all measurements. * $p < 0.05$, ** $p < 0.01$ and *** $p < 0.001$. All the results are represented as mean ± SEM.

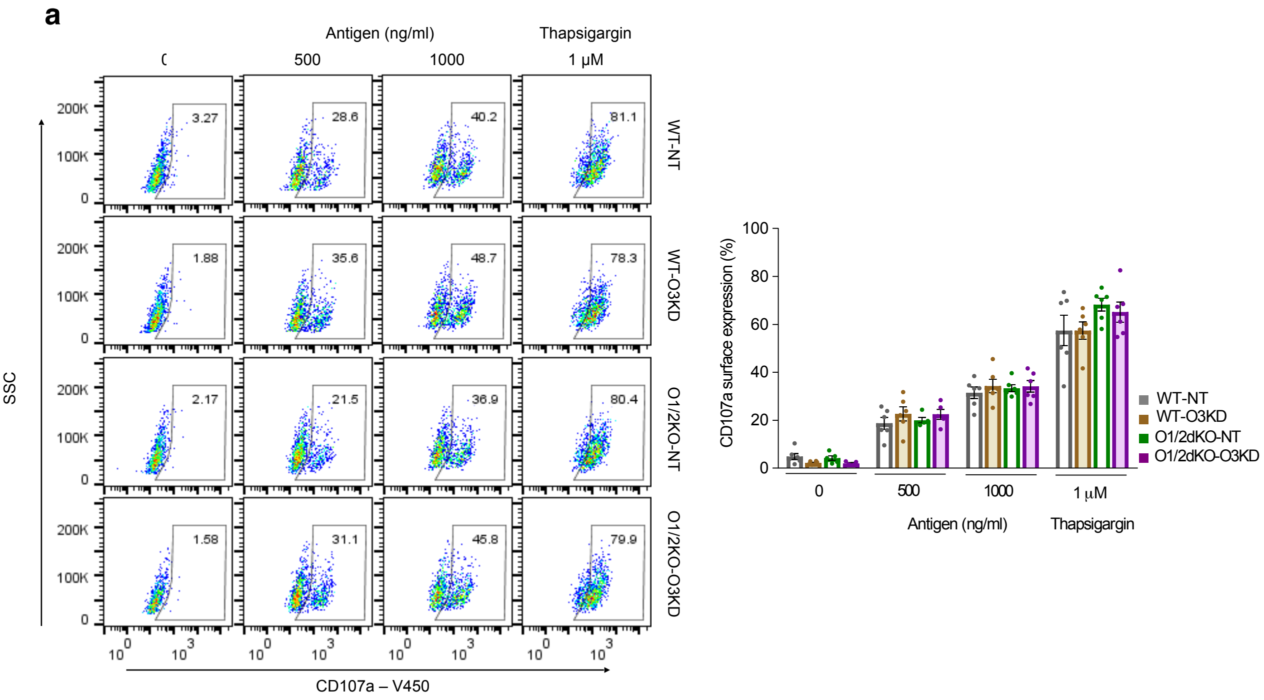

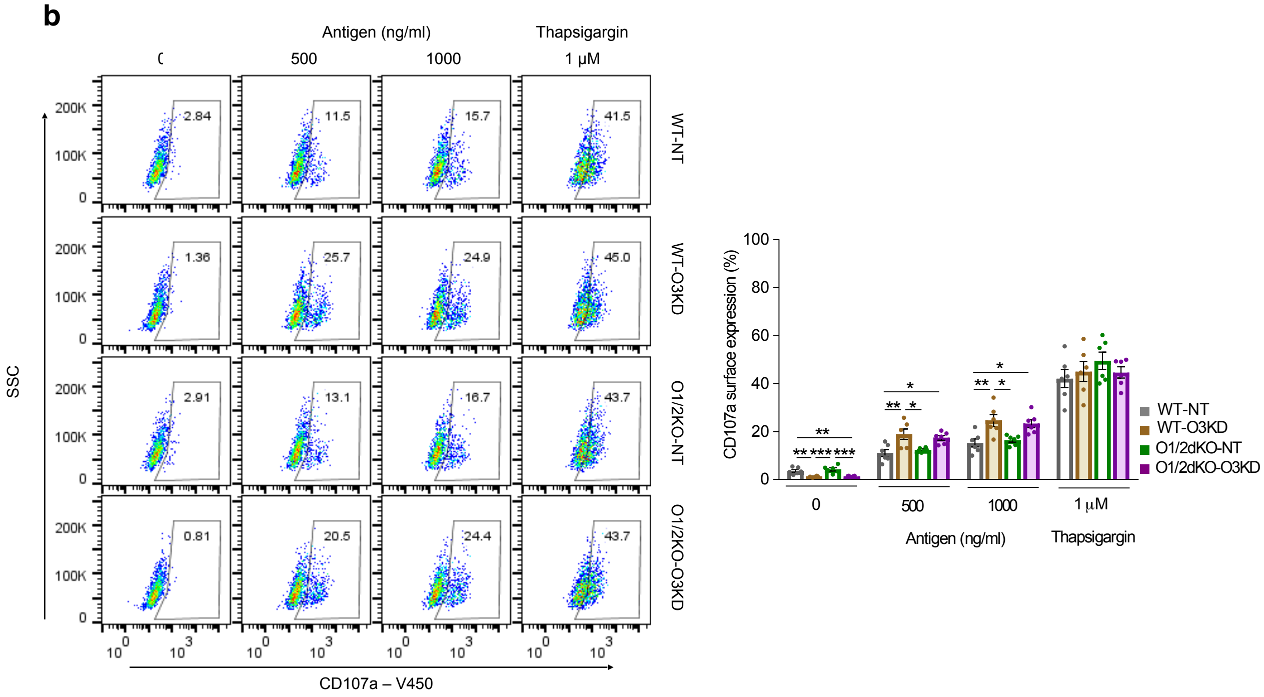

Supplementary Figure S3. Analysis of CD107a surface expression on BMMCs as a measure of degranulation. (**a**) Flow cytometric evaluation of CD107a following activation of BMMCs with 500 ng/ml and 1000 ng/ml TNP-BSA antigen or 1 μM thapsigargin for 30 minutes. (**b**) Flow cytometric evaluation of CD107a following activation of BMMCs with 500 ng/ml and 1000 ng/ml TNP-BSA antigen or 1 μM thapsigargin for 15 minutes. Data were analysed by one-way ANOVA with Tukey's posttest. n = 6 from 3 biological replicates and 2 independent experiments for all measurements. * $p < 0.05$, ** $p < 0.01$ and *** $p < 0.001$. All the results are represented as mean ± SEM.

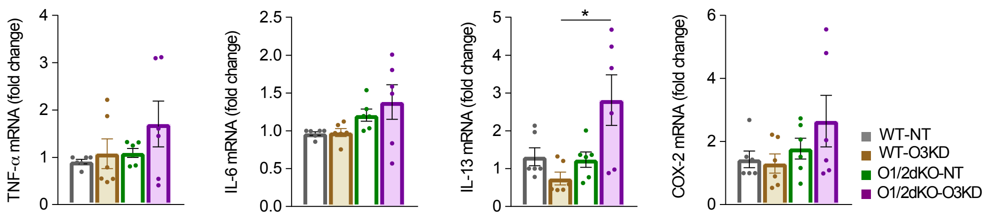

Supplementary Figure S4. Production of cytokines and COX-2 enzyme in unsensitised BMMCs. Relative mRNA expressions of pro-inflammatory cytokines TNF-α, IL-6 and IL-13 and COX-2 in unsensitised and non-activated BMMCs. Data were analysed using the Kruskal-Wallis test with Dunn's posttest, n = 6 biological replicates for all measurements. * $p < 0.05$. All the results are represented as mean ± SEM.

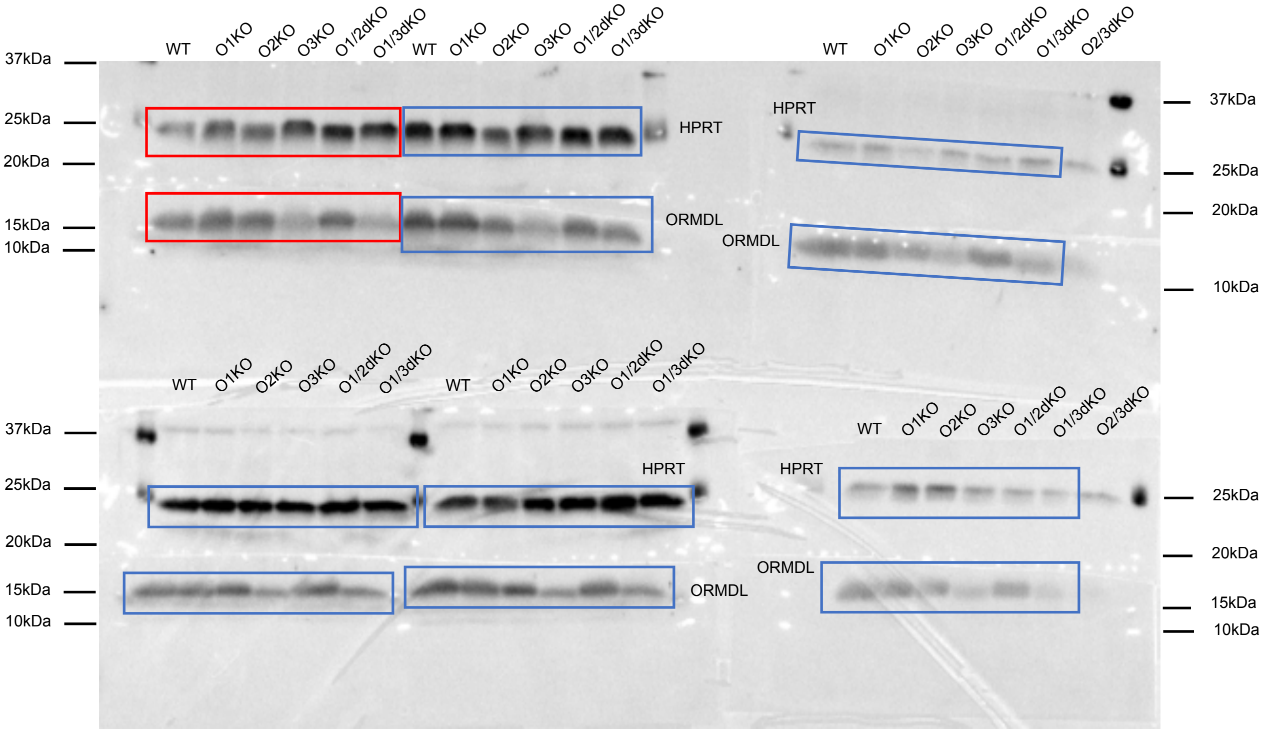

Supplementary Figure S5. Uncropped immunoblot images for Figure 1a.
Chemiluminescent images are overlaid with brightfield images of membranes for the visualisation of protein markers.
Red boxes indicate bands that were cropped for representative images. Blue boxes indicate additional bands that were used in statistical evaluation.

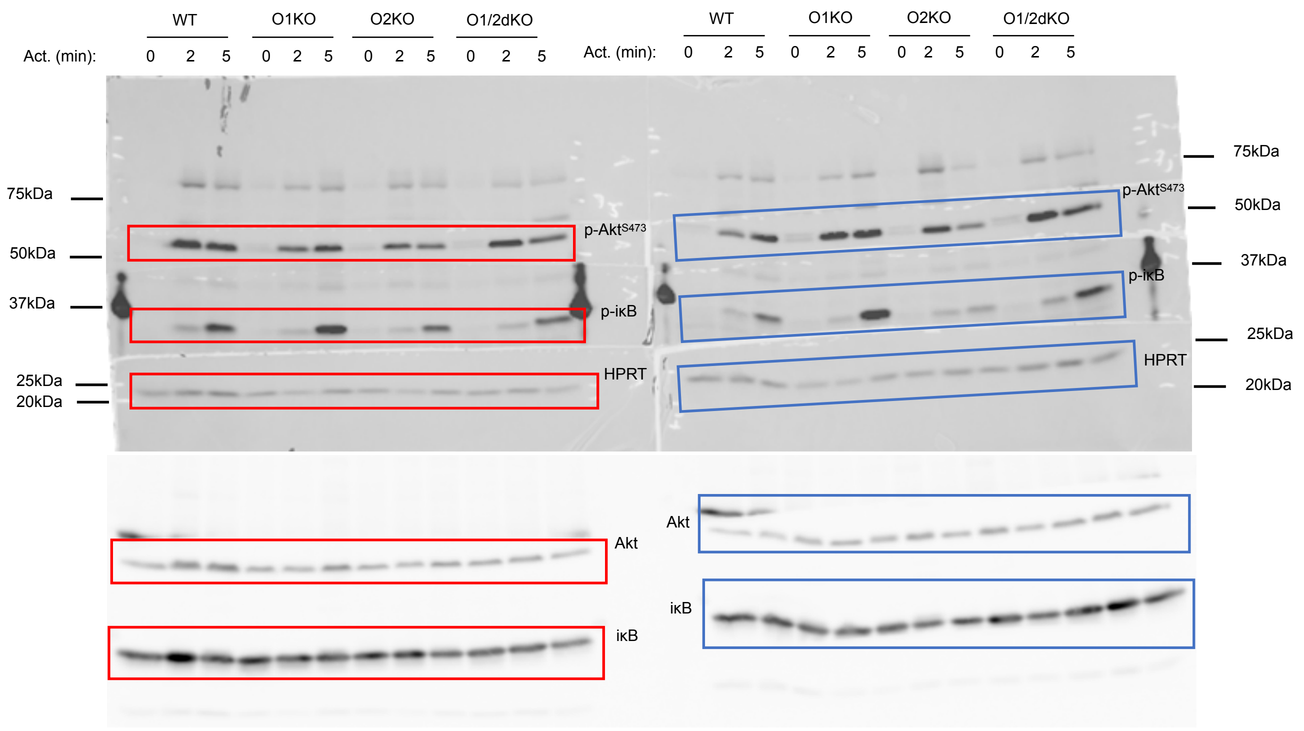

* No brightfield images for overlay were available for Akt and iκB loading bands

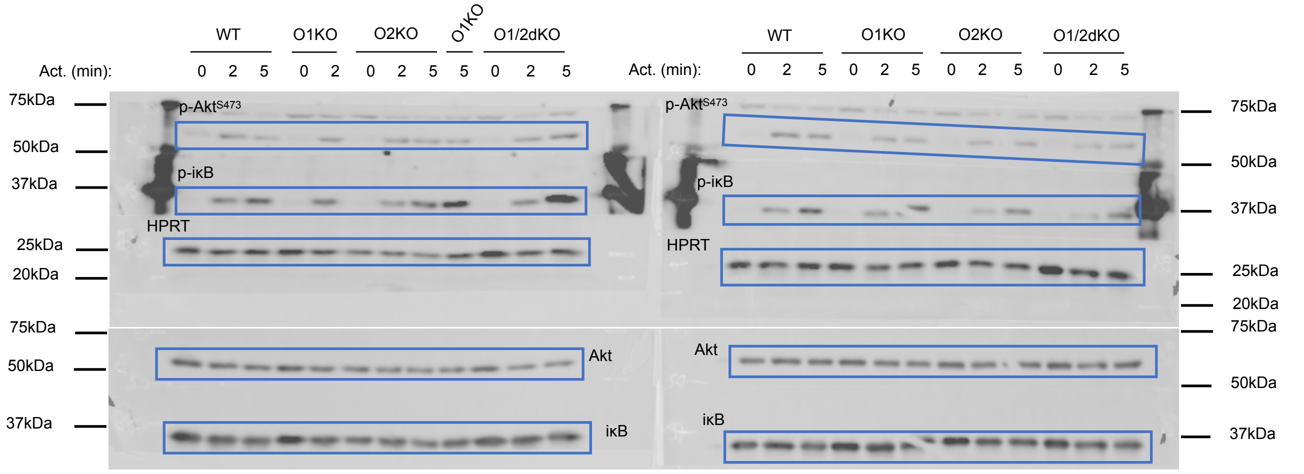

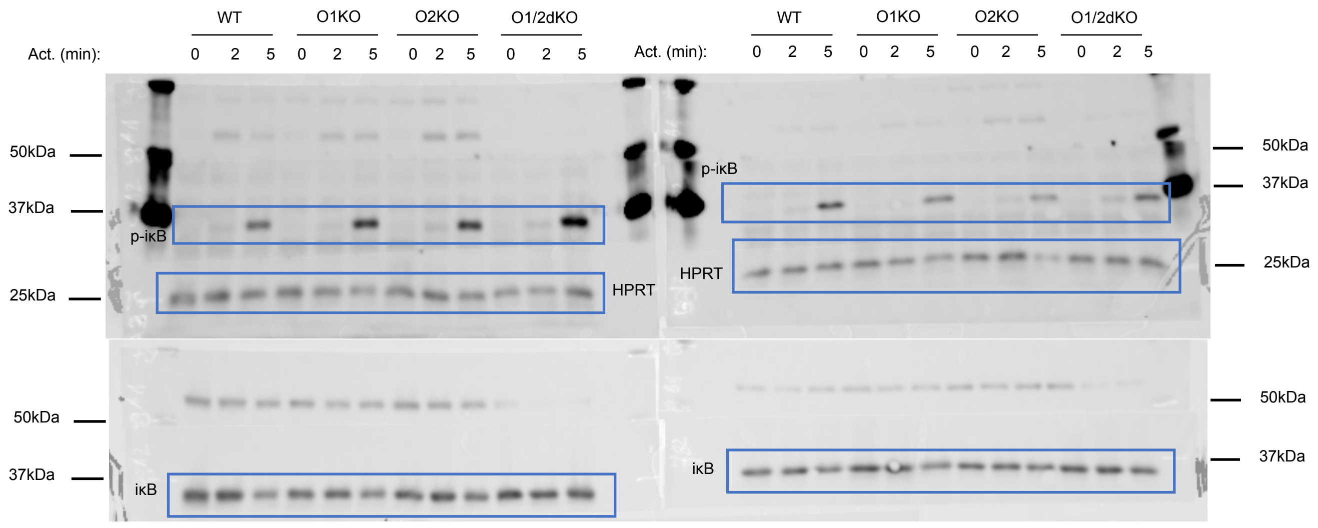

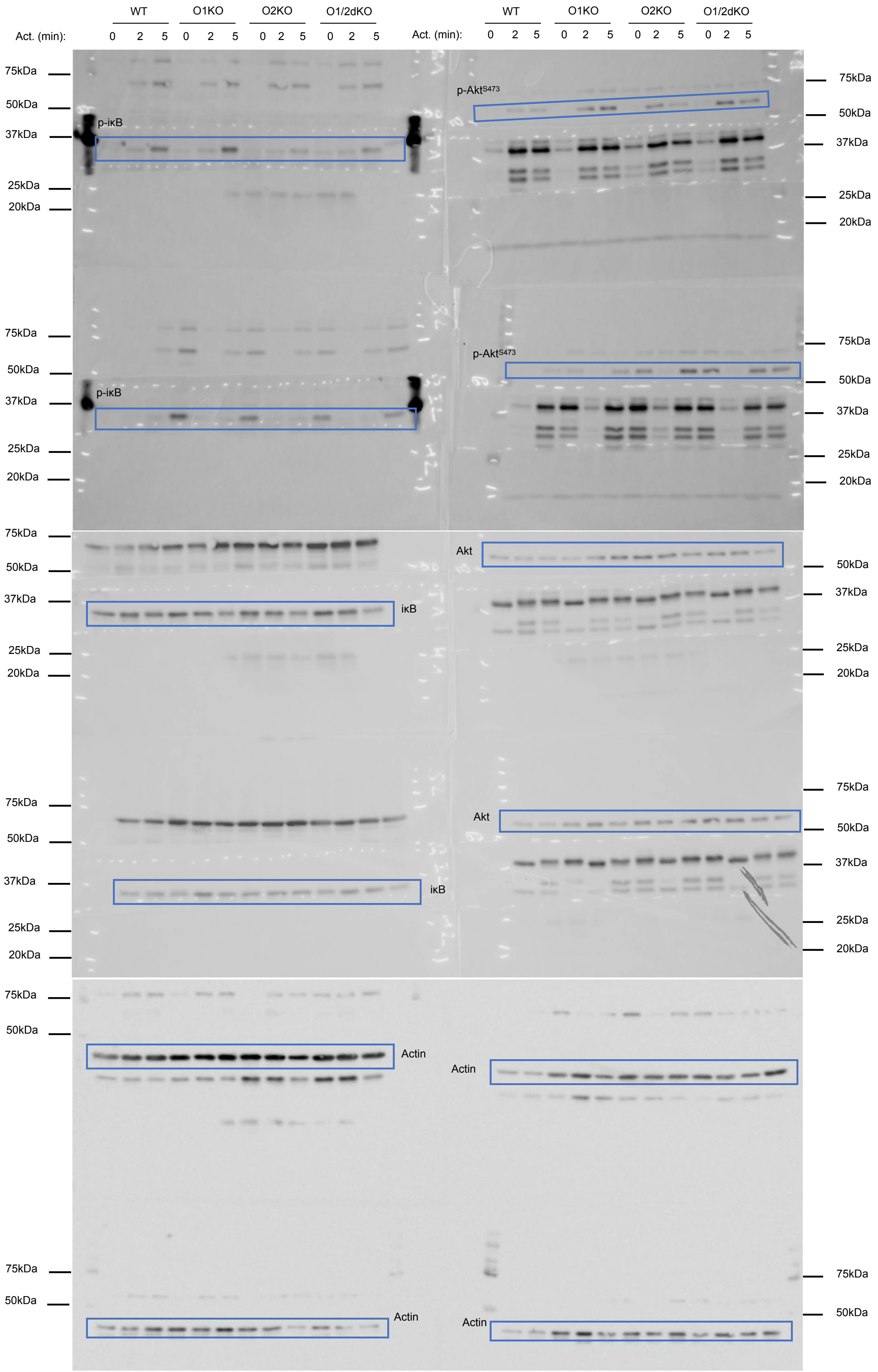
WT
O1KO
O2KO
O1/2dKO
Act. (min):
0 2 5 0 2 5 0 2 5 0 2 5
p-iκB
iκB
Actin
p-Akt^{S473}
Akt
75kDa
50kDa
37kDa
25kDa
20kDa

Supplementary Figure S6. Uncropped immunoblot images for Figure 1e.
Chemiluminescent images are overlaid with brightfield images of membranes for the visualisation of protein markers.
Red boxes indicate bands that were cropped for representative images. Blue boxes indicate additional bands that were used in statistical evaluation. Act. = activation.

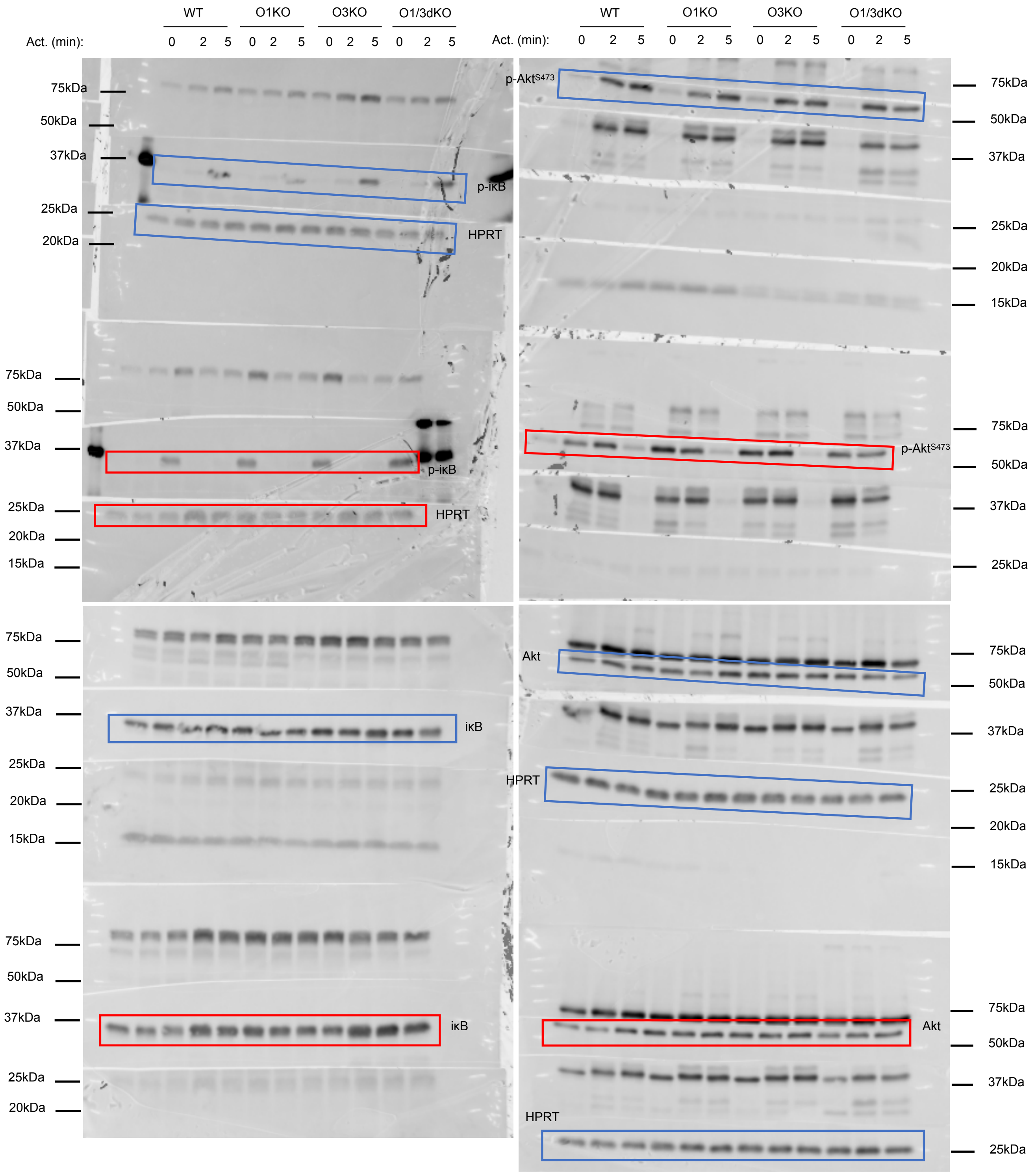
WT
O1KO
O3KO
O1/3dKO
Act. (min):
0 2 5 0 2 5 0 2 5 0 2 5
75kDa
50kDa
37kDa
25kDa
20kDa
15kDa
p-iκB
HPRT
iκB
p-Akt^{S473}
Akt

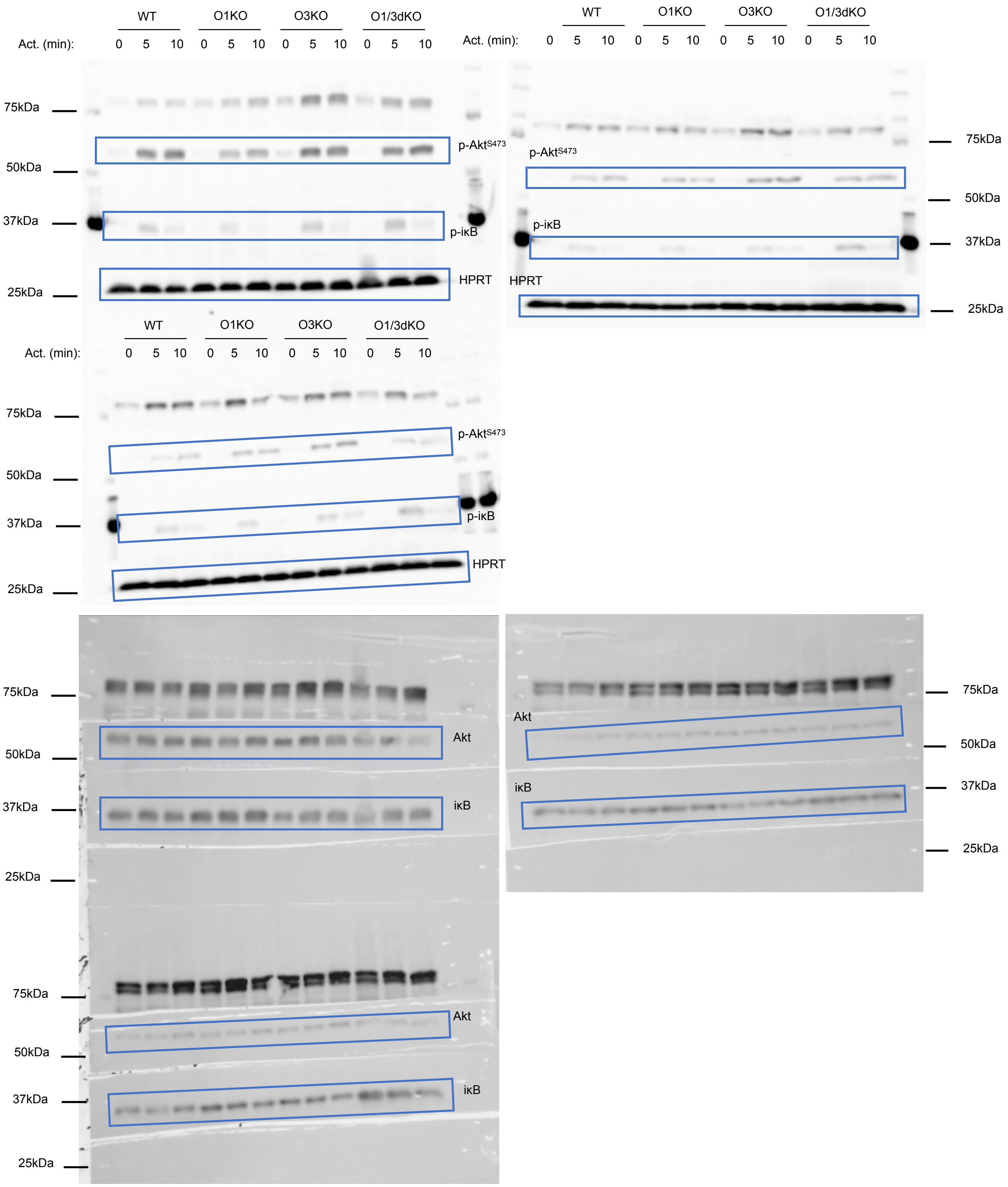

* Only protein bands from 0 and 5 minutes after antigen activation were used for statistical analysis

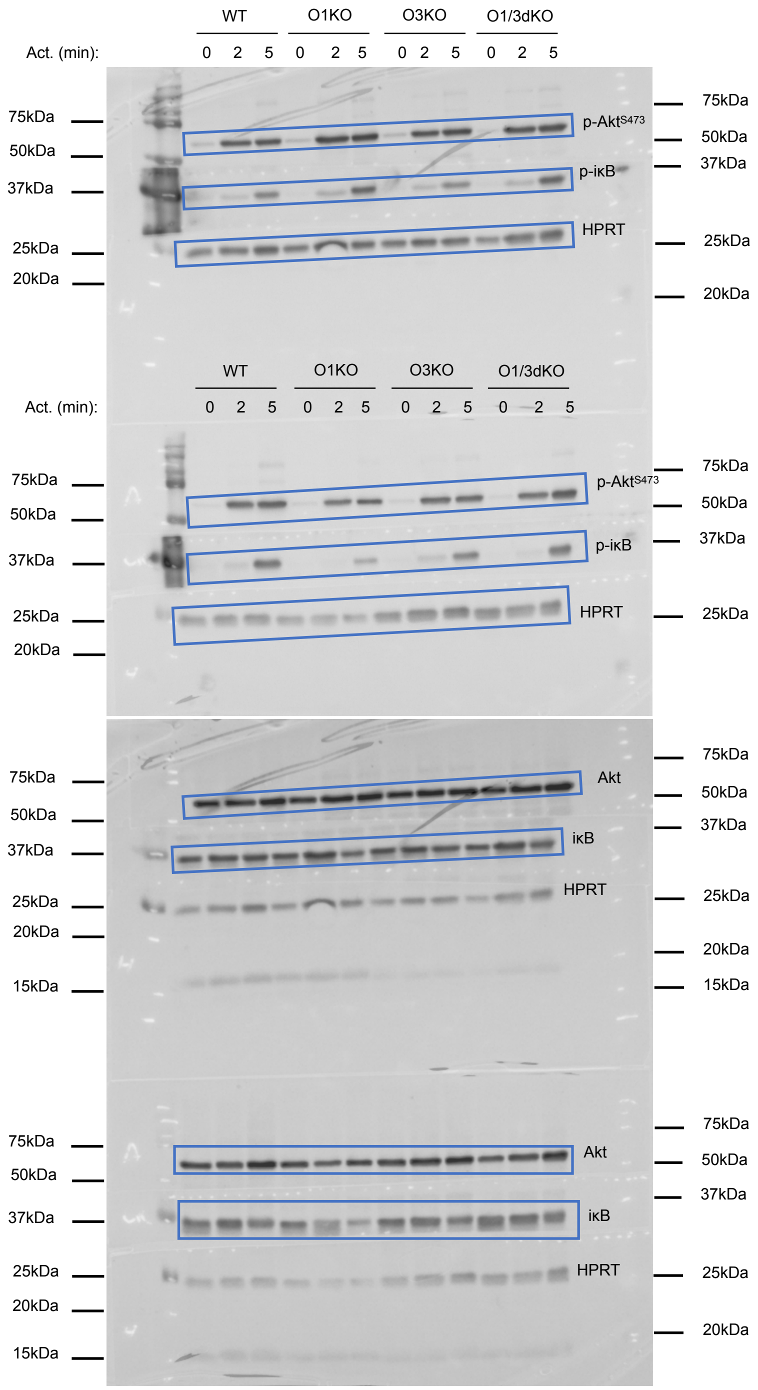

Supplementary Figure S7. Uncropped immunoblot images for Figure 1e.
Chemiluminescent images are overlaid with brightfield images of membranes for the visualisation of protein markers.
Red boxes indicate bands that were cropped for representative images. Blue boxes indicate additional bands that were used in statistical evaluation. Act. = activation.

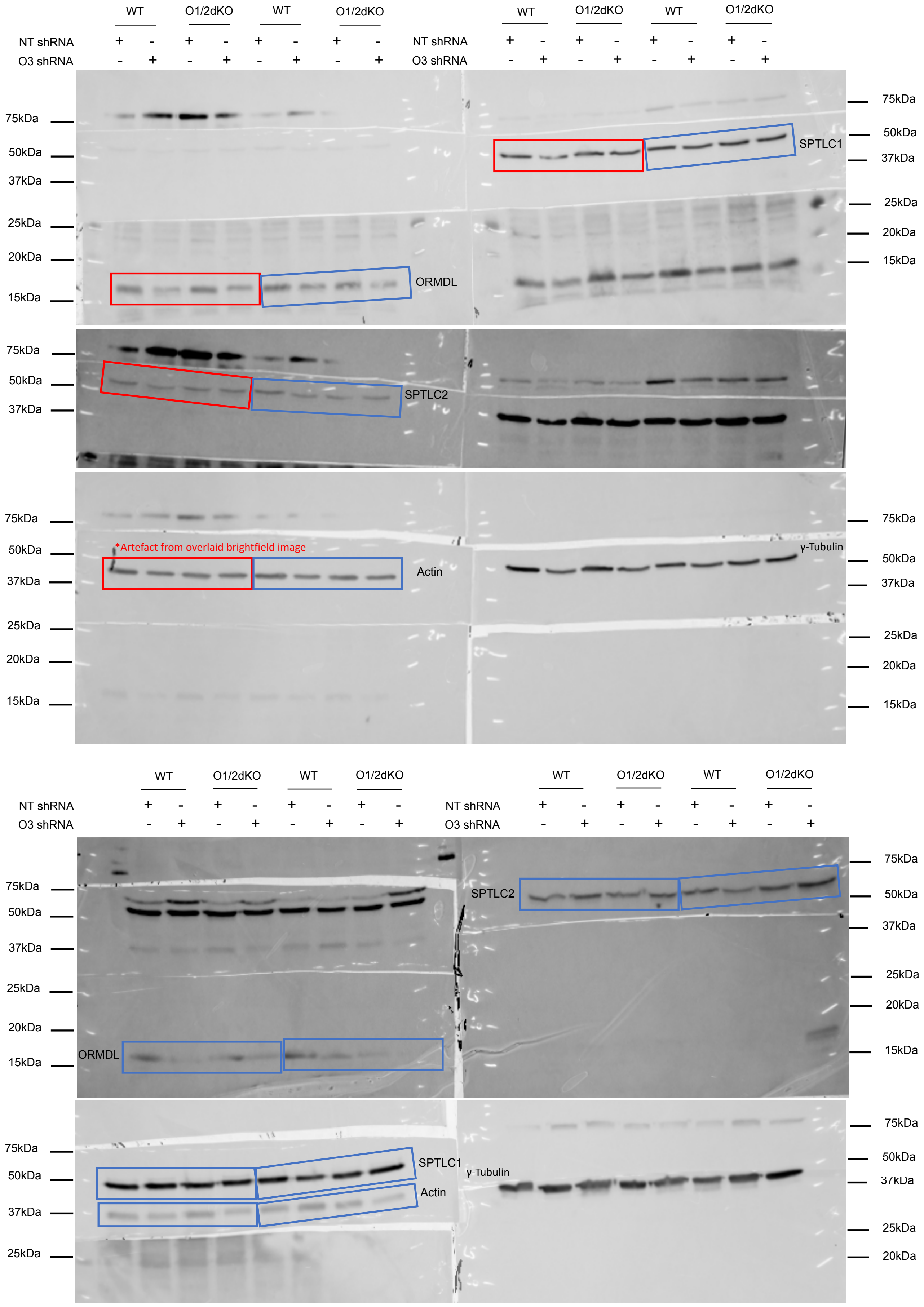

WT
O1/2dKO
WT
O1/2dKO
NT shRNA
O3 shRNA
75kDa
50kDa
37kDa
25kDa
20kDa
15kDa
SPTLC1
ORMDL
SPTLC2
*Artefact from overlaid brightfield image
Actin
γ-Tubulin

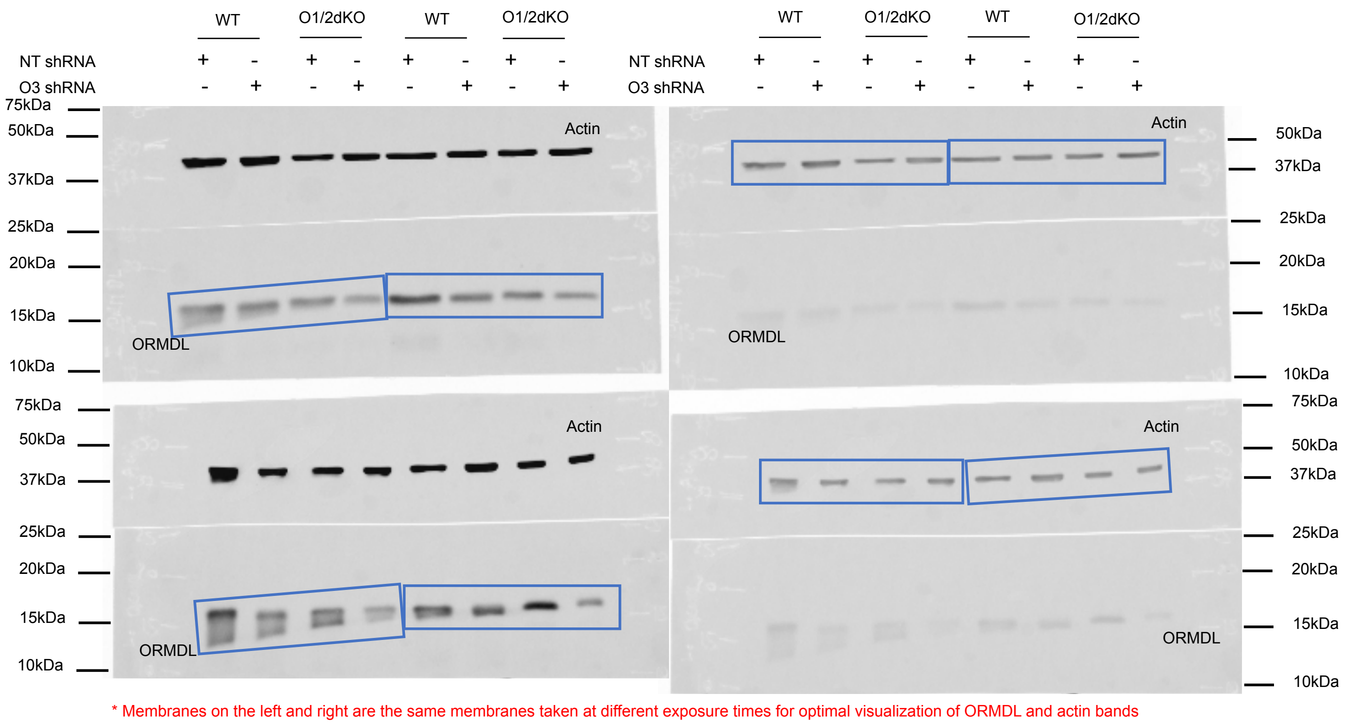

Supplementary Figure S8. Uncropped immunoblot images for Figure 2c.
Chemiluminescent images are overlaid with brightfield images of membranes for the visualisation of protein markers.
Red boxes indicate bands that were cropped for representative images. Blue boxes indicate additional bands that were used in statistical evaluation.

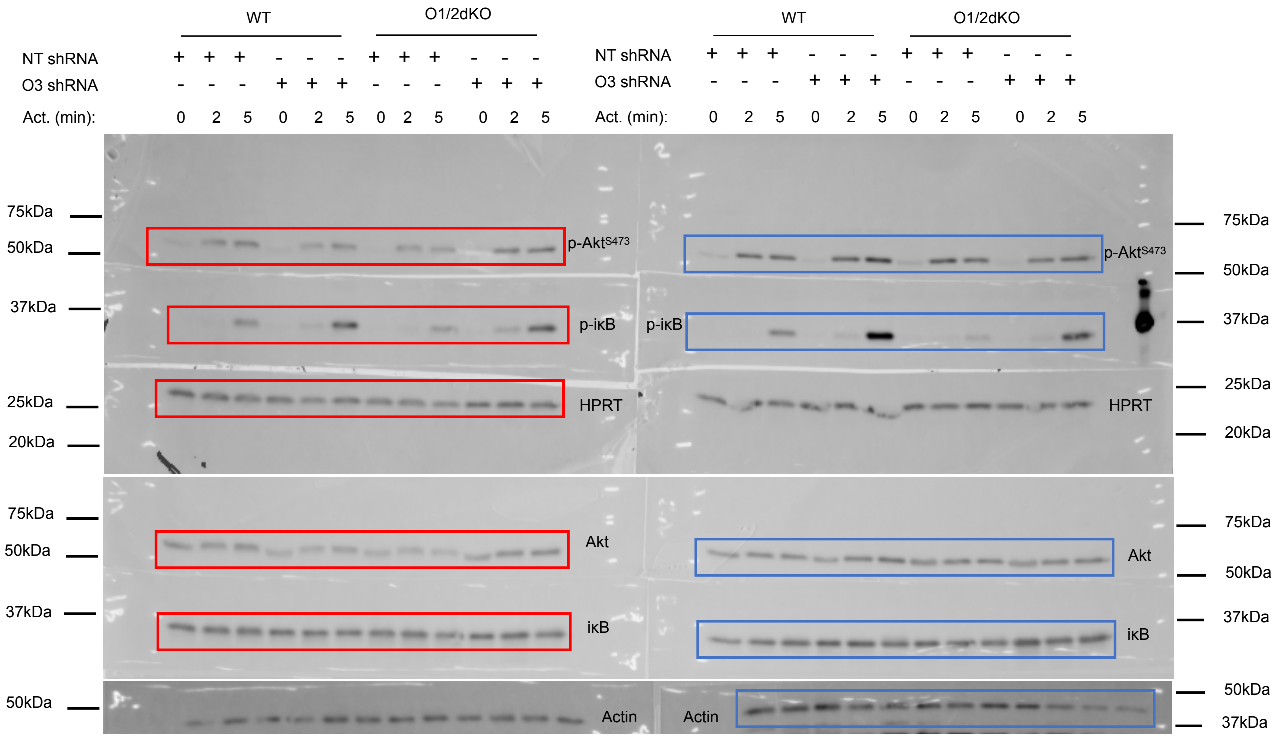
WT
O1/2dKO
NT shRNA
O3 shRNA
Act. (min):
75kDa
50kDa
37kDa
25kDa
20kDa
p-Akt S473
p-iκB
HPRT
Akt
iκB
Actin

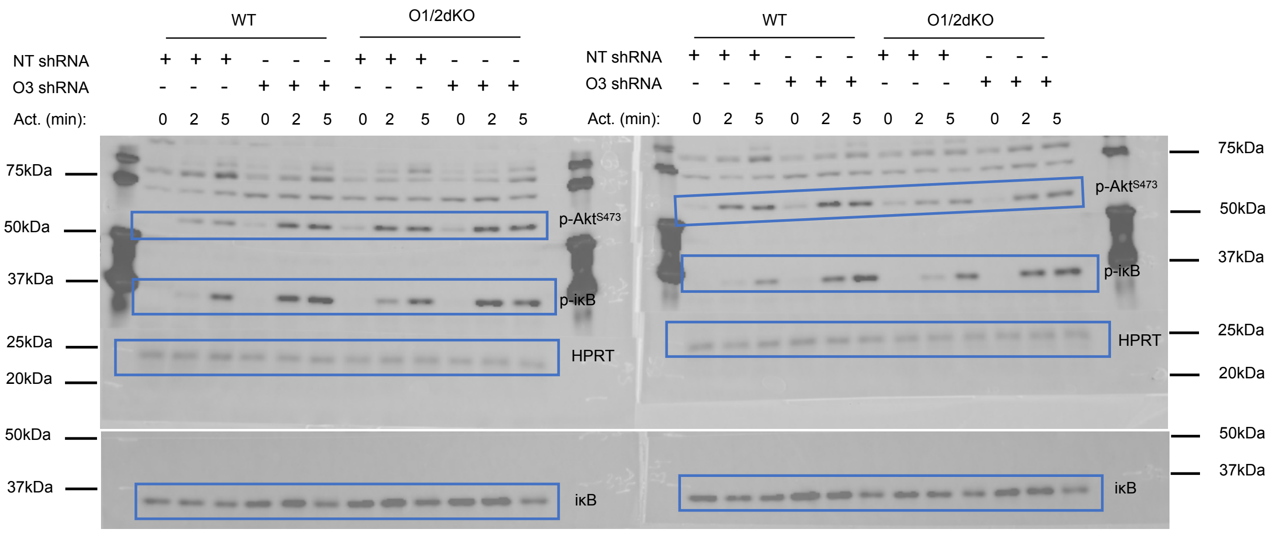
WT
O1/2dKO
NT shRNA
O3 shRNA
Act. (min):
75kDa
50kDa
37kDa
25kDa
20kDa
p-Akt S473
p-iκB
HPRT
iκB

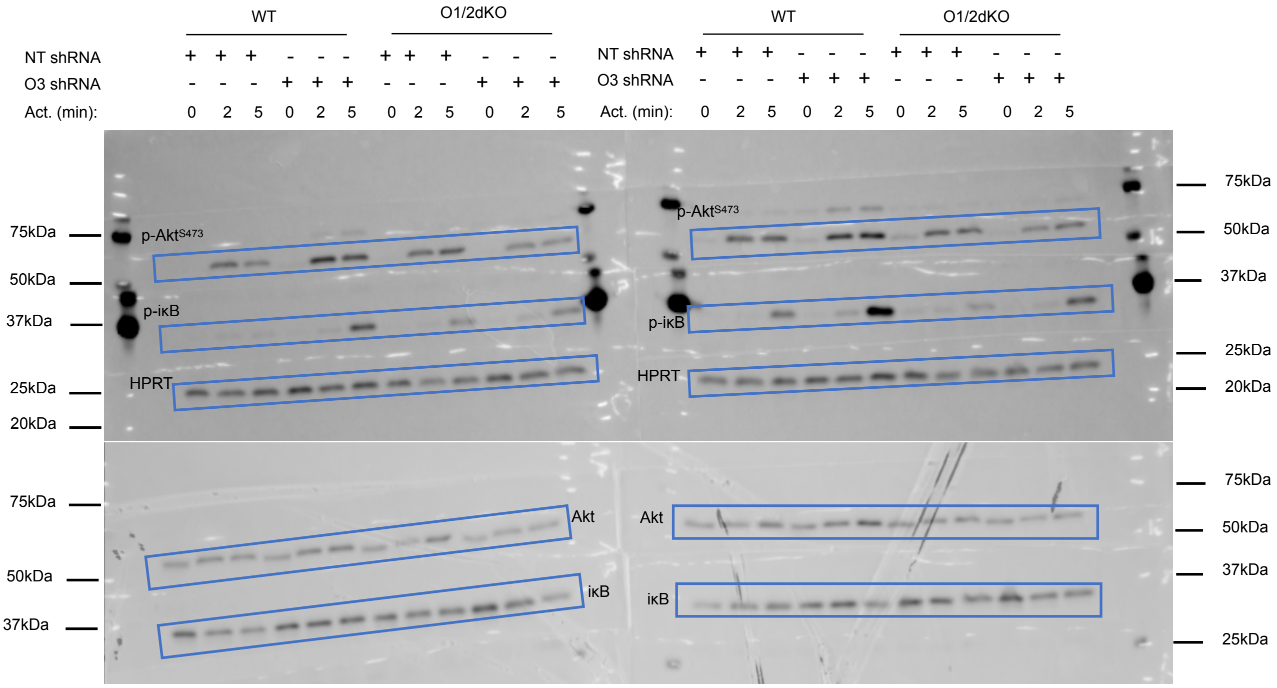

Supplementary Figure S9. Uncropped immunoblot images for Figure 4e.

Chemiluminescent images are overlaid with brightfield images of membranes for the visualisation of protein markers.

Red boxes indicate bands that were cropped for representative images. Blue boxes indicate additional bands that were used in statistical evaluation. Act. = activation.

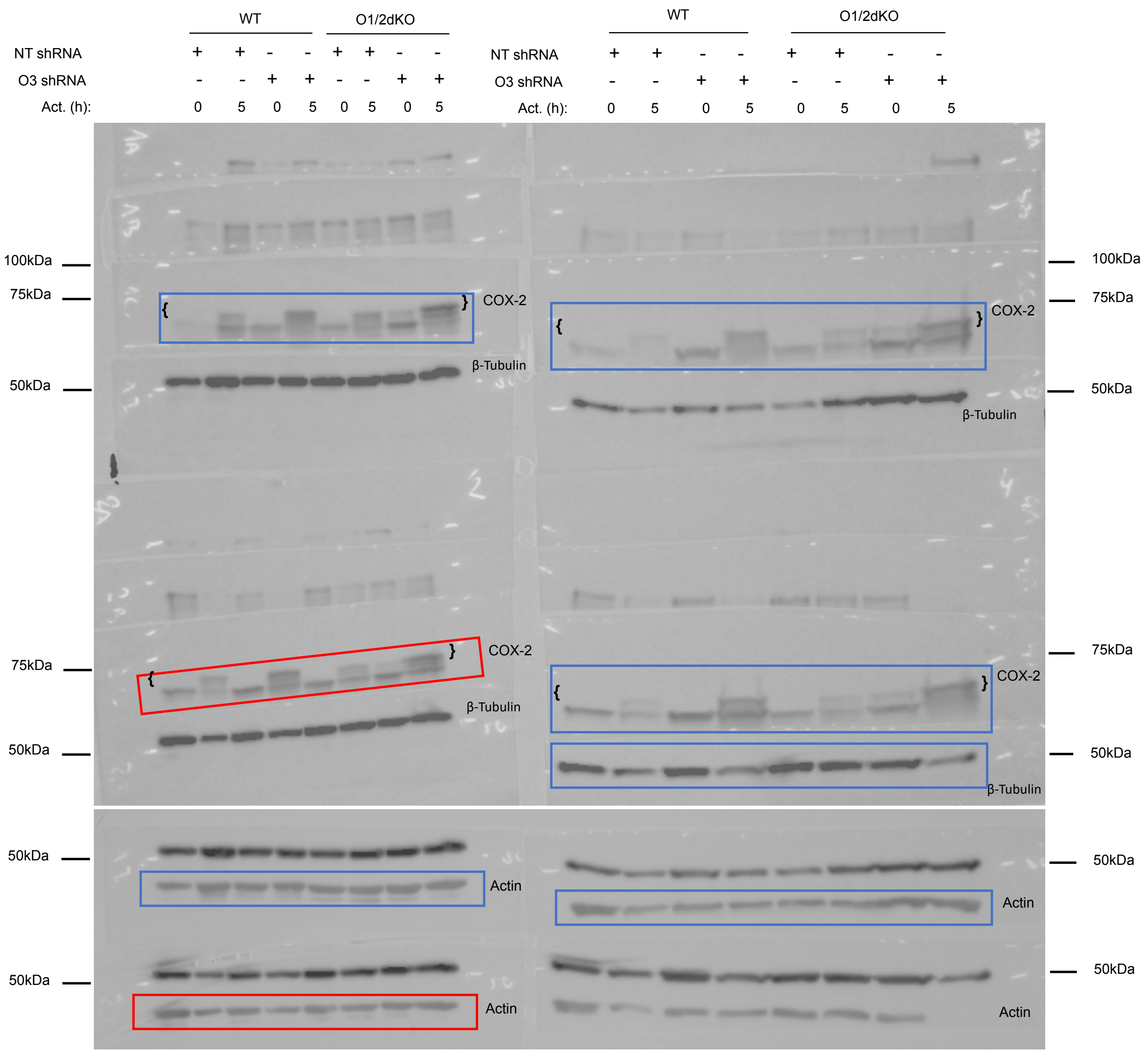

Supplementary Figure S10. Uncropped immunoblot images for Figure 6b.
Chemiluminescent images are overlaid with brightfield images of membranes for the visualisation of protein markers. Curly brackets indicate COX-2 protein bands (upper bands).
Red boxes indicate bands that were cropped for representative images. Blue boxes indicate additional bands that were used in statistical evaluation. Act. = activation.

Supplementary Table S1. Primary antibodies used in immunoblotting analysis.

Primary antibody	Clone/host	Supplier	Dilution	RRID
ORMDL	Polyclonal/rabbit	Our laboratory[1]	1:1000	N/A
SPTLC1	H-1/mouse	Santa Cruz Biotechnology	1:10000	AB_10917035
SPTLC2	Polyclonal/rabbit	Abcam	1:20000	AB_447617
Phospho-Akt (Ser473)	Polyclonal/rabbit	Santa Cruz Biotechnology	1:1000	AB_667741
Akt1	Polyclonal/goat	Santa Cruz Biotechnology	1:1000	AB_630849
Phospho-iκBα (Ser32)	14D4/rabbit	Cell Signaling Technology	1:1000	AB_561111
IκBα	L35A5/mouse	Cell Signaling Technology	1:1000	AB_390781
COX2	D5H5/rabbit	Cell Signaling Technology	1:1000	AB_2571729
HPRT1	F-1/mouse	Santa Cruz Biotechnology	1:400	N/A (Cat. Sc-376938)
β-actin	C4/mouse	Santa Cruz Biotechnology	1:5000	AB_2714189

1. Bugajev, V. *et al.* Negative regulatory roles of ORMDL3 in the FcεRI-triggered expression of proinflammatory mediators and chemotactic response in murine mast cells. *Cellular and molecular life sciences : CMLS* **73**, 1265-1285, doi:10.1007/s00018-015-2047-3 (2016).

Supplementary Table S2. Secondary antibodies used in immunoblotting analysis.

Secondary antibody	Host	Tag	Supplier	Dilution	RRID
Mouse IgG	Goat	HRP	Jackson ImmunoResearch Lab.	1:20000	AB_10015289
Rabbit IgG	Goat	HRP	Jackson ImmunoResearch Lab.	1:20000	AB_2307391
Goat IgG	Donkey	HRP	Santa Cruz Biotechnology	1:3000	AB_628490

Supplementary Table S3. Primers used for real-time quantitative PCR analysis.

Gene	Gene Bank Accession Number	Forward (5' to 3')<br>Reverse (5' to 3')	Amplicon (bp)
HPRT	NM_013556.2	CTGGTGAAAAGGACCTCTCGAA<br>CTGAAGTACTCATTATAGTCAAGGGCAT	110
UBB	NM_011664.4	ATGTGAAGGCCAAGATCCAG<br>TAATAGCCACCCCTCAGACG	160
TBP	NM_013684.3	GAAGAACAATCCAGACTAGCAGCA<br>CCTTATAGGGAACTTCACATCACAG	129
ORMDL1	NM_145517.4	TGGTATGTGGCTGACATATG<br>GCAAAAACACATACATCCCCAGA	134
ORMDL2	NM_024180.6	CACTCGAGTGATGAACAGT<br>AGGGTCCAGACAACAGGAATG	114
ORMDL3	NM_025661.4	CAACACACGGGTGATGAACAG<br>GACCCCGTAGTCCATCTGC	244
SPTLC1	NM_009269.2	ACGAGGCTCCAGCATACCAT<br>GGCCGGACACGATGTTGTAG	210
SPTLC2	NM_011479.4	TGCAGCACTCGTCAGGAAAT<br>CAGGCAACCTTTGCCAACAA	156
TNF-α	NM_013693.3	CCCTCACACTCAGATCATCTTCT<br>GCTACGACGTGGGCTACAG	61
IL-6	NM_031168.2	GAGGATACCACTCCCAACAGACC<br>AAGTGCATCATCGTTGTTCATACA	141
IL-13	NM_008355.3	AGACCAGACTCCCCTGTGCA<br>TGGGTCCTGTAGATGGATTG	123
COX-2	NM_011198.4	TGAGCAACTATTCCAAACCAGC<br>GCACGTAGTCTTCGATCACTATC	74
